# Breast cancer clinical outcomes and tumor immune microenvironment: cross-dialogue of multiple epigenetic modification profiles

**DOI:** 10.18632/aging.205853

**Published:** 2024-05-22

**Authors:** Chong Teng, Xiaowei Song, Chengjuan Fan, Siqi Man, Yuanyuan Hu, Yifei Hou, Tao Xin

**Affiliations:** 1 Department of Oncology, The Second Affiliated Hospital of Harbin Medical University, Harbin, Heilongjiang, China; 2 Oncology, Harbin Medical University, Harbin, Heilongjiang, China; 3 School of Nursing, Harbin Medical University, Harbin, Heilongjiang, China

**Keywords:** epigenetics, breast cancer, tumor metabolism, immunotherapy, methylation modification

## Abstract

The discovery of RNA methylation alterations associated with cancer holds promise for their utilization as potential biomarkers in cancer diagnosis, prognosis, and prediction. RNA methylation has been found to impact the immunological microenvironment of tumors, but the specific role of methylation-related genes (MRGs), particularly in breast cancer (BC), the most common cancer among women globally, within the tumor microenvironment remains unknown. In this study, we obtained data from TCGA and GEO databases to investigate the expression patterns of MRGs in both genomic and transcriptional domains in BC. By analyzing the data, we identified two distinct genetic groupings that were correlated with clinicopathological characteristics, prognosis, degree of TME cell infiltration, and other abnormalities in MRGs among patients. Subsequently, an MRG model was developed to predict overall survival (OS) and its accuracy was evaluated in BC patients. Additionally, a highly precise nomogram was created to enhance the practical usability of the MRG model. In low-risk groups, we observed lower TBM values and higher TIDE scores. We further explored how MRGs influence a patient’s prognosis, clinically significant characteristics, response to therapy, and the TME. These risk signatures have the potential to improve treatment strategies for BC patients and could be applied in future clinical settings. Moreover, they may also be utilized to determine prognosis and biological features in these patients.

## INTRODUCTION

Breast cancer (BC) is the leading cause of tumor-associated death in females worldwide and one of the most common malignancies. It is a highly heterogeneous disease, exhibiting various characteristics across different individuals. Owing to difficulties in early diagnosis and the aggressive nature of tumor progression, a significant number of BC patients are often diagnosed at an advanced stage or with metastatic lesions [1]. As a result of early detection and treatment, 5-year relative survival rate was almost 100% for BC patients diagnosed primarily in stage I, compared to only 26% for BC patients initially diagnosed in stage IV [2]. According to PAM50, BC has five subtypes (i.e., luminal A, luminal B, Her2-enriched, normal-like, and basal-like) [3]. However, it has been observed that patients with the same molecular type and similar clinical characteristics can exhibit different prognoses and responses to chemotherapy or immunotherapy [4], suggesting other subtle factors affecting their prognosis and treatment outcome. Prognosis in BC patients can always be improved with an accurate diagnosis, detection, and treatment [5]. Hence, to enhance the prognosis and treatment of BC patients, it is essential to investigate new effective biomarkers and therapy modalities. The post-transcriptional modifications have attracted much attention in BC research because its important role in BC biology [2].

Messenger RNA (mRNA) and non-coding RNA are post-transcriptional modifications [6]. To date, numerous studies have identified more than 170 RNA modifications [7]. RNA modification disorders have been linked to a wide range of diseases, including BC [8]. Post-transcriptional alterations include N6-methyladenosine (m^6^A), 5-methylcytosine (m^5^C) and N1-methyladenosine (m^1^A), which affect adenosine in various ways [6]. m^6^A is the most common variation in mRNA, initially found in the 1970s [9]. It has been described to be a changeable, normal adenosine alteration, particularly abundant in mammalian mRNA 3 ’UTR [10]. In addition, m^6^A has been found to be associated with various cellular processes such as RNA stabilization, metabolism, transcriptional control, and intracellular signaling. Its biological significance can be seen in several contexts, including embryonic self-renewal, the development of hematopoietic stem/progenitor cells, circadian rhythm regulation, heat shock response, neurofunction, and cancer. These examples highlight the diverse roles that m6A plays in essential biological processes [8]. m^5^C is a common human RNA mutation found in both mRNAs and ncRNAs [11]. So far, the human genome has been revealed to have overall m^5^C sites. This reversible post-transcriptional modification affects many molecular processes, including RNA-protein interactions, RNA stability, and translation efficiency [8]. It follows orders by “author” (methyltransferase), “reader” and “eraser” (demethylase). According to a recent study, NSUN6 mediates location-specific deposition of m^5^C in mRNA to regulate translation quality [12]. It is known that the m^5^C modification can be transformed into various kinds of alterations, such as hm^5^C, although the m^5^C demethylase is unclear [13]. In recent years, abnormal m^5^C has been discovered to be carcinogenic in several malignancies [8]. Preliminary results of m^1^A were available more than 50 years ago. Adenosine becomes m^1^A when a methyl group joins the N1 site. The Watson-Crick base pair interface is the location of the methyl group, disrupting the base pair, while the positive charge carried by m^1^A affects local RNA structure or protein-RNA interactions [10]. M^1^A is also found to be a highly enrichment effect for 5 ’UTR translation [14]. Based on earlier studies, M^1^A was found in tRNA, mitochondrial transcripts, as well as rRNA and mRNA [15]; In eukaryotes, m^1^A is abundant in tRNA and rRNA. Furthermore, recent studies have revealed that m^1^A alteration may be impacts mRNA translation [16].

Cancer-related RNA methylation alterations have been recognized as a promising alternative for developing biomarkers that can be used in prediction, prognosis, and diagnosis. Discoveries of methylation associated with antitumor immunity are promising [17]. Methylation, like necrosis, is an important cellular response that regulates cancer progression, spread, and metastasis [6]. However, the mechanism that methylation modulators affect the prognosis, and the underlying molecular pathways of BC is not well understood. methylation landscapes help predict BC prognosis, and in recent years STOX1, UNKL, ZMAT3, and ZNF443 have all been identified as novel biomarkers of BC metastasis with strong methylation deregulation and association with metastasis [18]. Although methylation is critical for both oncogenic and anticancer pathways, few studies have addressed the significance of methylation in cancer, particularly in BC.

Cancer immunotherapy uses anti-tumor immune responses to identify and destroy tumor cells by stimulating the host immune system [19]. T cell-related immune responses only partially benefit a small percentage of cancer patients. Although T cells contribute to immune checkpoint inhibitors, they also stimulate anti-cancer responses [20, 21]. BC is known to be an immunologically dormant tumor, which slows obviously its response to immunotherapy. Encouraging preclinical trial results, along with years of clinical data, suggest that immunotherapies may hold the key to ushering breast cancer into an era of clinical intervention that has yet to be experienced [22]. Furthermore, there is growing evidence that immune infiltration in the tumor microenvironment (TME) plays an important role in determining BC progression. According to a large number of studies, the prognosis of patients with triple-negative BC was related to the quantity of T cells obviously in the tumor [23]. For this reason, the method we got from immune cell infiltration (ICI) profiles—clustering of BC samples relied on molecularly specific subgroups related to the ICI patterns—may be the most reliable and promising way to thoroughly assess tumor sensitivity to immunotherapy. It also facilitates personalized treatments to increase their effectiveness. But the investigation did not think over the incomplete context of BC’s TME, because it was mediated by ICI patterns.

With the rapid advancement of science, our research on cancer treatment, prognostic biomarkers, and the carcinogenic framework will continue to progress. We aim to investigate the prognostic significance, expression, and presentation of the regulatory axis of MRGs in BC. Our findings have the potential to offer further insights into the molecular mechanisms and prognostic biomarkers associated with BC.

## MATERIALS AND METHODS

### Data sources

Clinicopathological information and RNA-seq data on BC were collected from The Cancer Genome Atlas (TCGA; https://portal.gdc.cancer.gov/) and Gene Expression Omnibus (GEO; https://www.ncbi.nlm.nih.gov/geo/) databases. The expression profiles of mRNA were acquired from the Ensembl database (http://asia.ensembl.org) following the addition of GTF file-based annotations. We included 1001 BC patients from TCGA-BRCA in our subsequent analysis because we removed data from individuals with incomplete or less than 30 days of overall survival (OS) information. The 88 BC patients from GSE20711 were used as a dataset for external validation. The clinical information of breast cancer patients was shown in Supplementary Table 5.

### Consensus clustering analysis of MRGs

Supplementary Table 2 contains information for these 57 MRGs. Using the R package “Consensus Cluster Plus,” consensus unsupervised clustering analysis was carried out to group patients into various molecular subtypes based on MRG expression. The following criteria were used to classify these items: the cumulative distribution function curve first developed smoothly and steadily. Second, no group had a sample size that was too small. Thirdly, despite a drop in the inter-group correlation, the intra-group correlation rose.

### Correlation between molecular subtypes with the clinical features and prognosis of BC

Kaplan-Meier curves were used to compare OS among the two MRG clusters, which was generated by the “survival” and “survminer” R programs. The two different MRG clusters showed a significant difference in the MRG transcriptional profile. Patients with decreased MRG cluster revealed significant therapeutic advantages and clinical benefits. In addition, we evaluated the association between genetic subtypes, clinicopathological traits, and prognosis to explore the clinical utility of the two clusters through consensus clustering. HER2, PR, ER, age, gender, tumour site, TNM stage, subtypes, and clinical stage were patient characteristics.

### Relationship of molecular subtypes with TME and evaluation of the immune status

Gene set variation analysis (GSVA) was utilized to examine the changes in MRGs in biological processes. The single sample gene set enrichment analysis was used to assess the infiltration of distinct immune cells (ssGSEA) [24]. Using the R package “clusterprofiler,” we conduct functional enrichment analysis on the differentially expressed genes (DEGs), allowing us to more completely investigate the hidden functions of the methylation clusters DEGs and discern the enriched pathways as well as gene functions that go along with them.

### Construction of the prognostic risk model

First, univariate Cox regression analysis was applied to DEG. Next, patients were categorized into different subtypes (gene cluster A, B and C) utilizing the prognostic DEGs for unsupervised clustering. To create a predictive model, we randomly created training and testing sets at a ratio of 0.7:0.3. LASSO Cox regression analysis was simply applied to prognostic DRGs to reduce the likelihood of overfitting. We use risk score = Σ (expi * coefi) to determine the risk score, (expi and coefi stand for each gene’s expression and risk coefficient). Using the median risk score, we classified patients into high-risk and low-risk groups. We also assessed differences in risk scores for MRG clusters and gene clusters. Risk score accuracy was assessed applying Kaplan-Meier analysis and receiver operating characteristic (ROC) curves. Using the “rms” program and the outcomes of the independent prognosis analysis, the clinical characteristics and risk score were combined to create a prediction nomogram.

### Mutation, immunotherapy response, and drug susceptibility analysis

Infiltrating immune cells were assessed for high- and low-risk groups using CIBERSORT. The 22 immune cells’ relationships with the risk score and the genes in the model are examined. The TCGA database generates a mutation annotation format in order to identify somatic mutations in several BC sample groupings. To evaluate the tumor component in each sample, we computed the tumor purity, stromal, immunological, and CIBERSORT scores using the CIBERSORT algorithm. We determine the TBM score for each BC patient across all categories. We investigated the connections between various social groups and TIDE. We generated the usage semi-inhibitory concentration (IC50) values of a pRRophetic package of anti-tumor pharmaceuticals for BC to examine the differences in the therapeutic effects of regularly used anti-tumor medications between the two groups.

### Cell culture and infection

BC cells MDA-MB-231 and MCF7 were provided by Dr. Chen and cultured under 5% CO2. After overnight incubation, the cells were transfected with small interfering RNA (siRNA) using lipo2000 (Invitrogen, USA) or Lipofectamine RNAiMax transfection reagent (Invitrogen), according to the manufacturer’s instructions. The targeting siRNA was synthesized by Genepharma (Shanghai, China) and RiboBio (Guangzhou, China). The transfected cells were identified by quantitative real-time PCR (qRT-PCR), using the primer sequences provided in Additional File 1 and Supplementary Table 1.

### Apoptosis detection

Assessment of cell apoptosis was carried out using flow cytometry. For this purpose, cells were first harvested prior to their resuspension in 100 μl of 1 x binding buffer. This was followed by the addition of 5 μl of fluorescein isothiocyanate (FITC), annexin V and propidium iodide (PI) (556,547; BD Biosciences, USA), with the cell suspension subsequently incubated for 15 min at room temperature. Eventually, the samples were attenuated with 400 ul of 1 x binding buffer prior to analysis with an ACS Calibur flow cytometer.

### Immunohistochemistry

59 pairs of breast cancer tissues were retrieved from Department of Pathology, the Second Affiliated Hospital of Harbin Medical University from March to June 2014. The study was approved by the Institutional Review Board of our hospital (No. KY2024-001). And all patients signed informed consent forms.

IHC was performed based on streptavidin and peroxidase method. Staining scores were shown as mean optical density (MOD), which was assessed by the software image-Pro Plus 6.0 Image. And the cytoplasmic staining of ZMAT3 (1:500; 10504-1-AP; Thermo Fisher Scientific, USA) was indicated as positive. Based on the value of MOD, the clinical features of breast cancer patients were analyzed.

### Statistics

The data processing in our investigation was handled by R and GraphPad software. The Wilcoxon test was used to compare and analyze the characteristics of the two groups, and Kruskal-Wallis test was used to compare and analyze the profiles of more than two groups. The R script was shown in Supplementary File 1.

### Data availability statement

The datasets presented in this study can be found in online repositories. The names of the repository/repositories and accession number(s) can be found in the article/Supplementary Material.

## RESULTS

### Genetic and transcriptional alterations of MRGs in BC

**Figure 1:**
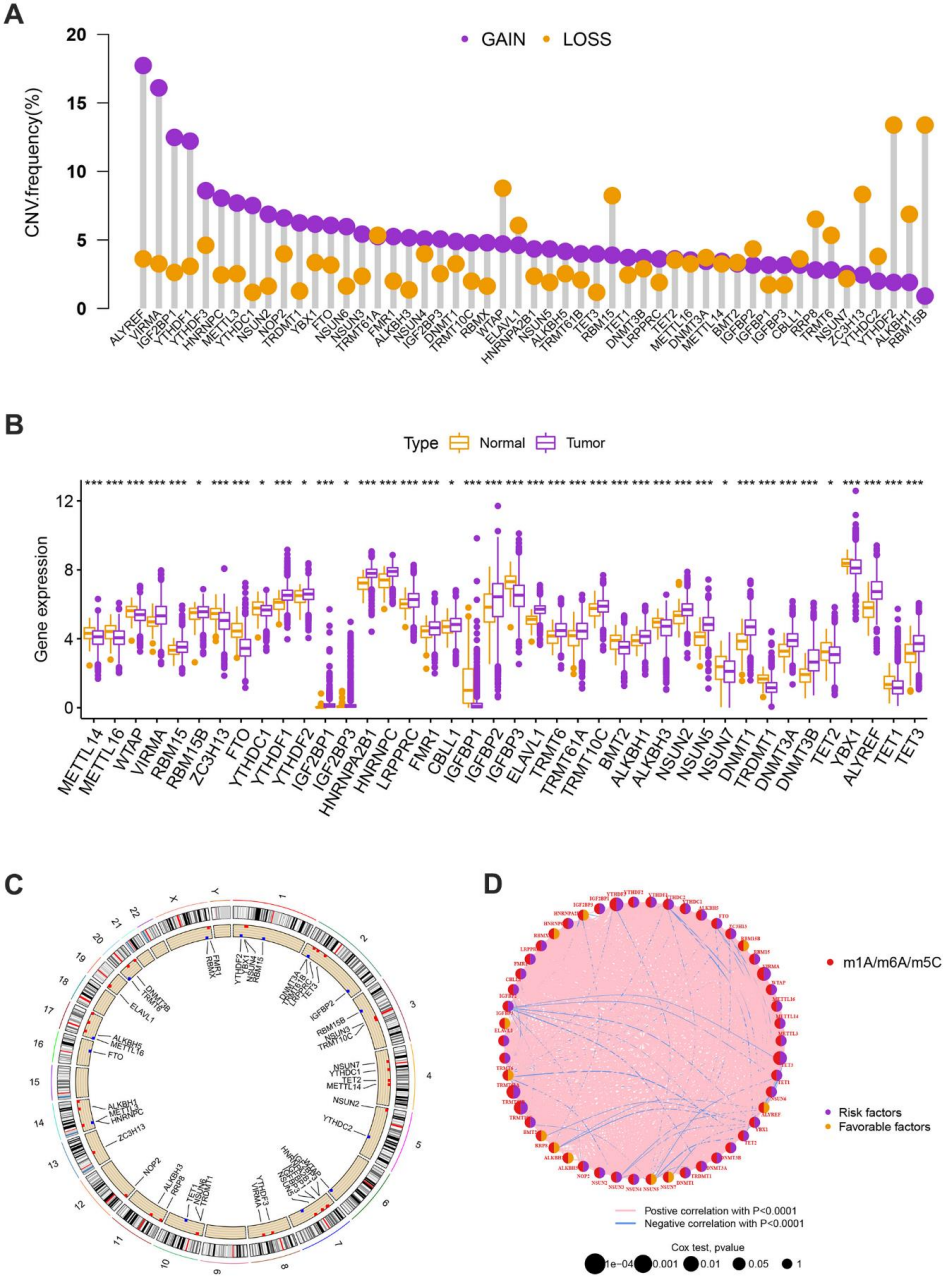
(**A**) The CNV of 57 MRGs. (**B**) Expression distributions of differentially expressed MRGs between normal and BC tissues. Orange: the normal breast tissues; purple: breast cancer tissues. (**C**) The positions of the CNV alterations on their respective chromosomes for these MRGs. (**D**) The overall group of MRG interactions, regulatory factor connectivity, and value of prognosis in BC patients were identified in the network. The circle size reflects the P-value. The purple circle reflects the risk factors while the orange circle reflects the favorable factors. The pink line is represented by positive correlation with P<0.0001 while the blue line is represented by negative correlation with P<0.0001.

Significant differences in the expression levels and genetic make-up of MRGs were found between the BC and control samples, indicating that MRGs may have diverse functions in the initiation and progression of BC carcinogenesis. The 57 MRGs’ somatic copy number variation was the next area of investigation. Most CNV modifications were concentrated on the amplificated copy number, but RBM15B, ALKBH1, ZC3H13, RBM15, and WTAP, which had a high frequency of CNV deletion, according to the analysis of CNV modification frequency (Figure 1A). MRGs with CNV gain were expressed at higher levels, ALYREF, VIRMA, IGF2BP1, YTHDF1, and others showed considerably higher expression in BC tissues compared to normal tissues (Figure 1B). Figure 1C displayed the location of CNV alteration of MRGs on chromosomes. The experiments stated above revealed the incredibly varied topography of genetic and expressional variation in MRGs between normal, suggesting the possibility that CNV regulates the mRNA expression of MRGs. It hints that the dysregulated expression of MRGs plays an important role in the carcinogenesis and development of BC.

### Identification of methylation-related subtypes

**Figure 2:**
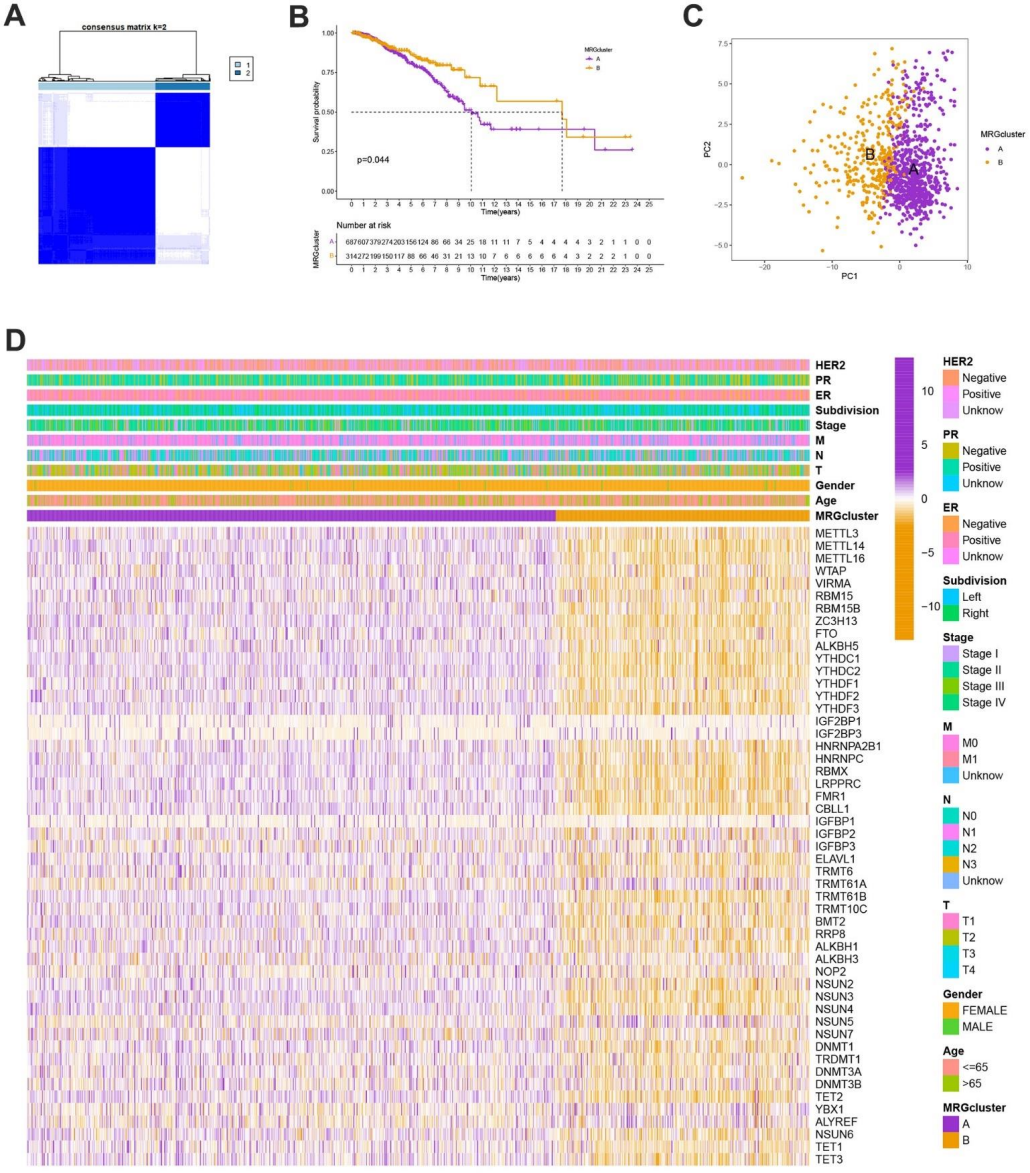
(**A**) Consensus matrix heatmap defining A and B MRG clusters (k = 2). (**B**) Kaplan-Meier curves indicated a shorter OS in patients with MRG cluster A than that in patients with MRG cluster B. P-values calculated by log-rank test. (**C**) The principal component analysis of A and B MRG clusters. (**D**) Heatmap of the clinical features and MRGs expression levels between the two MRG clusters. Colors from orange to purple indicate the trend of MRGs expression levels from low to high.

We selected 1001 patients to further examine the expression pattern of MRG linked to carcinogenesis. The methylation network demonstrated the importance of methylation in BC patients, regulatory factor connectivity, and the general group of MRG connections (Figure 1D). Based on the expression profiles of the 57 MRGs, we used a consensus clustering method to categorize the BC patients. We found that the entire cohort was the best choice for MRG clusters A and B based on k = 2. (Figure 2A). Patients in MRG cluster B had a better OS, hinted by the Kaplan-Meier curves (p =0. 044; Figure 2B). In BC analysis, the transcriptional profiles of MRGs were different clearly between the two subtypes (Figure 2C). In addition, we demonstrate significant differences in MRG expression and pathology characteristics (Figure 2D).

### Evaluation of TME

**Figure 3:**
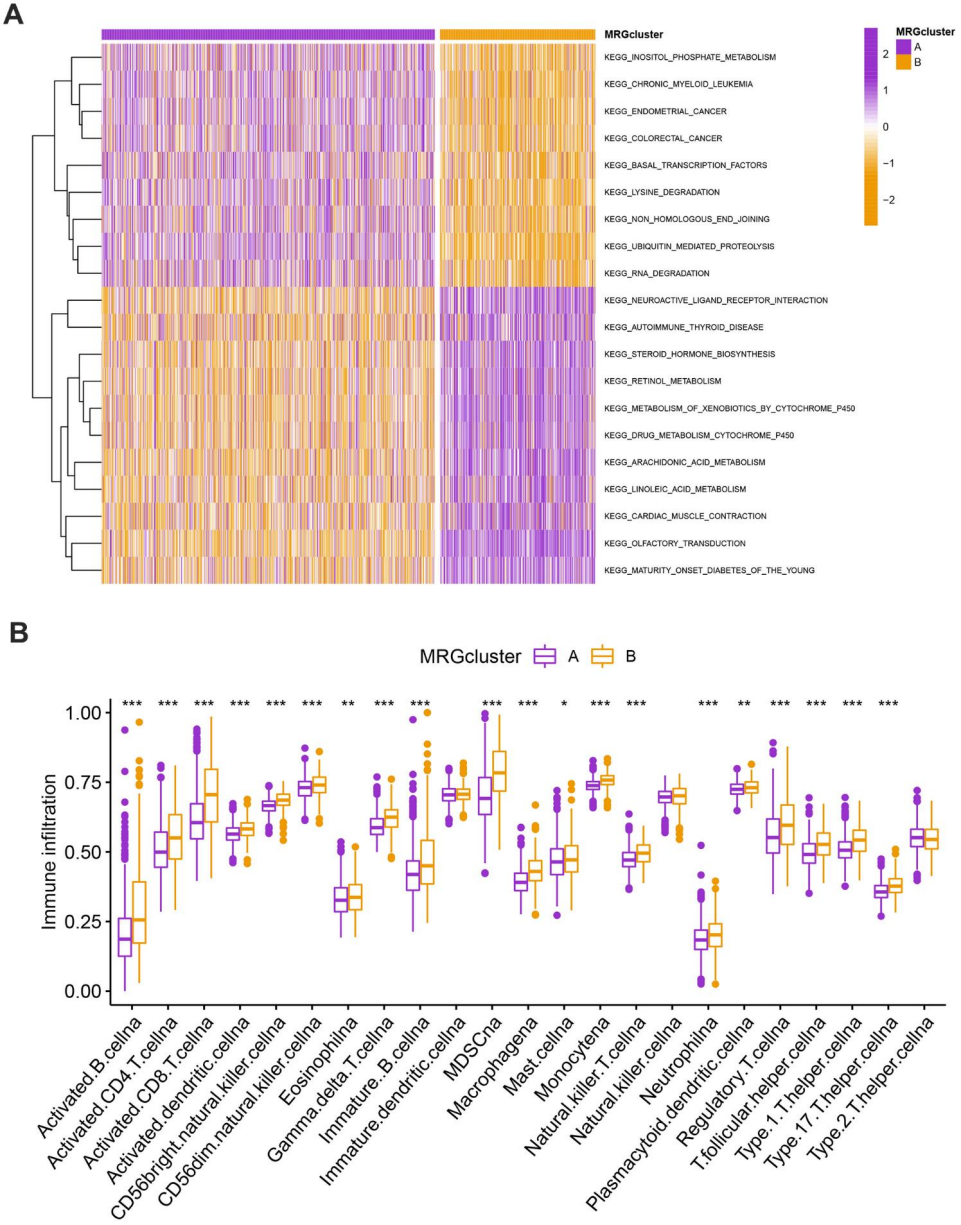
(**A**) Heatmap of significantly enriched KEGG subset of canonical pathways by GSEA analysis in A and B MRG clusters. (**B**) The infiltration levels of distinct immune cells in A and B MRG clusters were evaluated by ssGSEA analysis. *P<0.05, **P<0.01, ***P<0.001.

The GSVA enrichment analysis revealed a significant difference between MRG clusters B and A. One of them focuses on inositol phosphate metabolism, chronic myeloid leukemia, endometrial cancer, and colorectal cancer, while the other one is maturity-onset diabetes of the young, olfactory transduction, cardiac muscle contraction, and linoleic acid metabolism (Figure 3A and Supplementary Table 3). We look into the relationships between the 22 different subsets of human immune cells and the two subtypes of each BC sample using the CIBERSORT method. There were considerable changes in the invasion patterns of immune cells between the two kinds (Figure 3B).

### Classification of gene clusters

**Figure 4:**
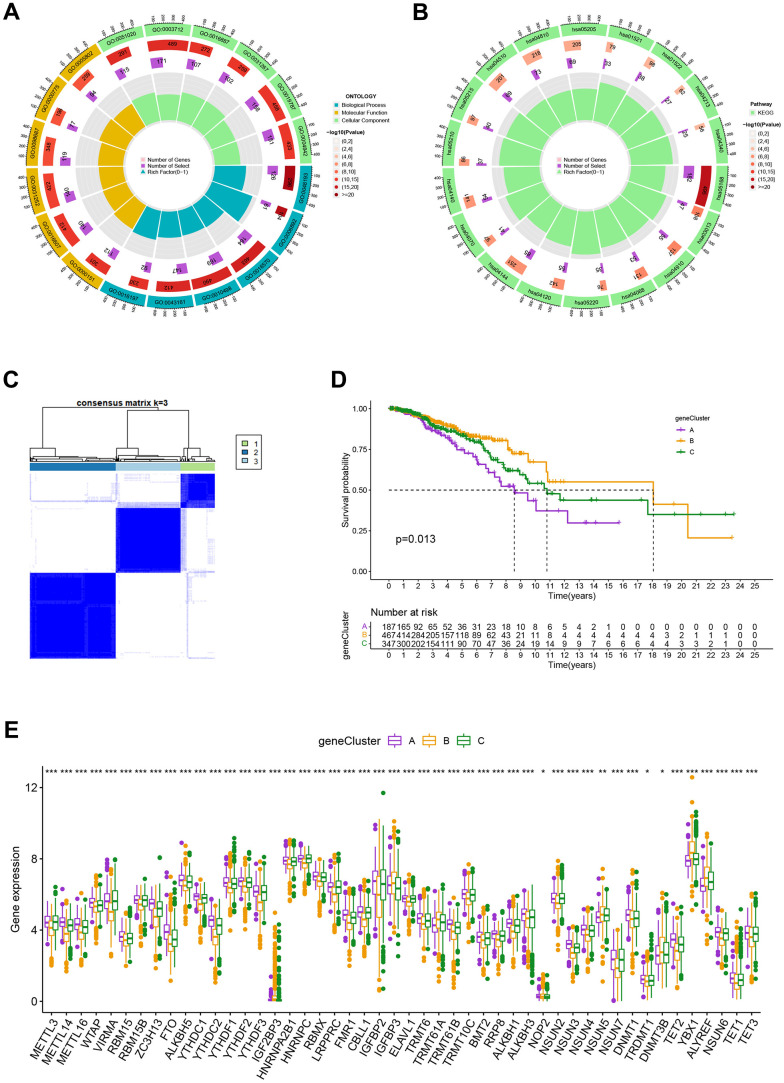
(**A**, **B**) GO and KEGG enrichment analyses. (**C**) Consensus matrix heatmap defining A, B and C gene clusters (k = 3). (**D**) Kaplan-Meier curves indicated that patients in gene cluster B had higher OS compared with patients in gene cluster A and C. P-values calculated by log-rank test. (**E**) The difference of gene expression levels between the three gene clusters. *P<0.05, **P<0.01, ***P<0.001.

**Figure 5:**
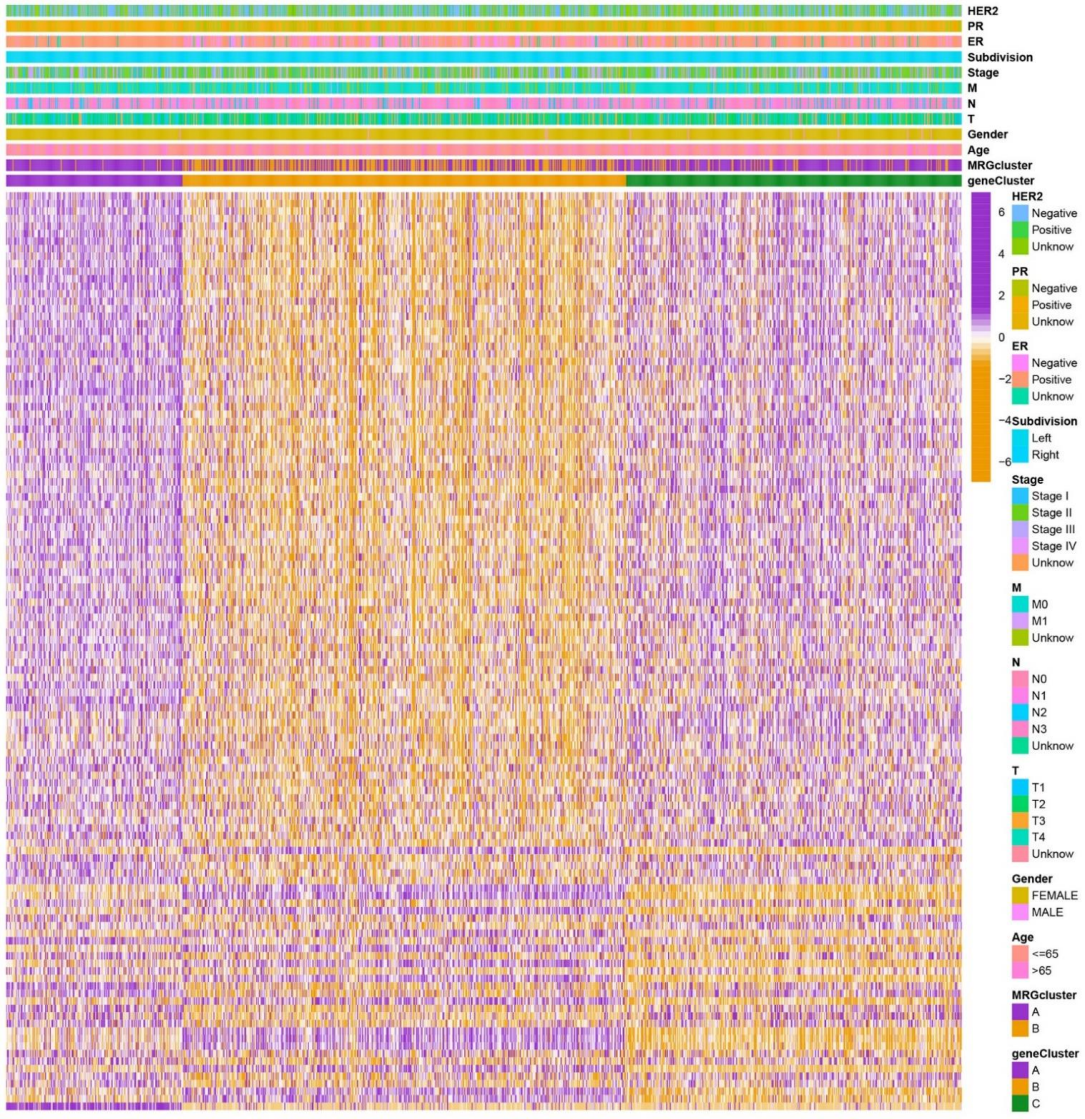
**Heatmap of the clinical features and gene expression levels between the three gene clusters.** Colors from orange to purple indicate the trend of gene expression levels from low to high.

To investigate the biological behavior that underpins each focused flash pattern, 4097 DEGs associated with MRG cluster were found using the R package “limma”. Functional enrichment analysis was then carried out on this data (Figure 4A, 4B). These DEGs were involved in immunity and were present during all biological activities (Figure 4A and Supplementary Table 4). By demonstrating that cancer- and immunological-related pathways were enriched, KEGG analysis demonstrated the significance of methylation in the immune regulation of the TME (Figure 4B and Supplementary Table 4). Using univariate Cox regression analysis, 122 genes related to OS duration were chosen ((p<0.01). In order to validate these regulatory mechanisms, patients were divided into three gene categories based on prognostic genes using consensus clustering methodologies. Based on k = 3, we conclude that the entire cohort was the best option for gene clusters A, gene clusters B, and gene clusters C (Figure 4C). According to Kaplan-Meier curves (p<0. 013; Figure 4D), patients with gene cluster B had the highest OS, which is better than that of cluster A and cluster C. The three gene clusters’ MRG expression varied significantly, which was in line with our assumptions (Figure 4E). A study of the clinicopathological characteristics of several gene clusters also uncovered a large diversity in clinical characteristics (Figure 5).

### Construction and validation of the prognostic risk model

**Figure 6:**
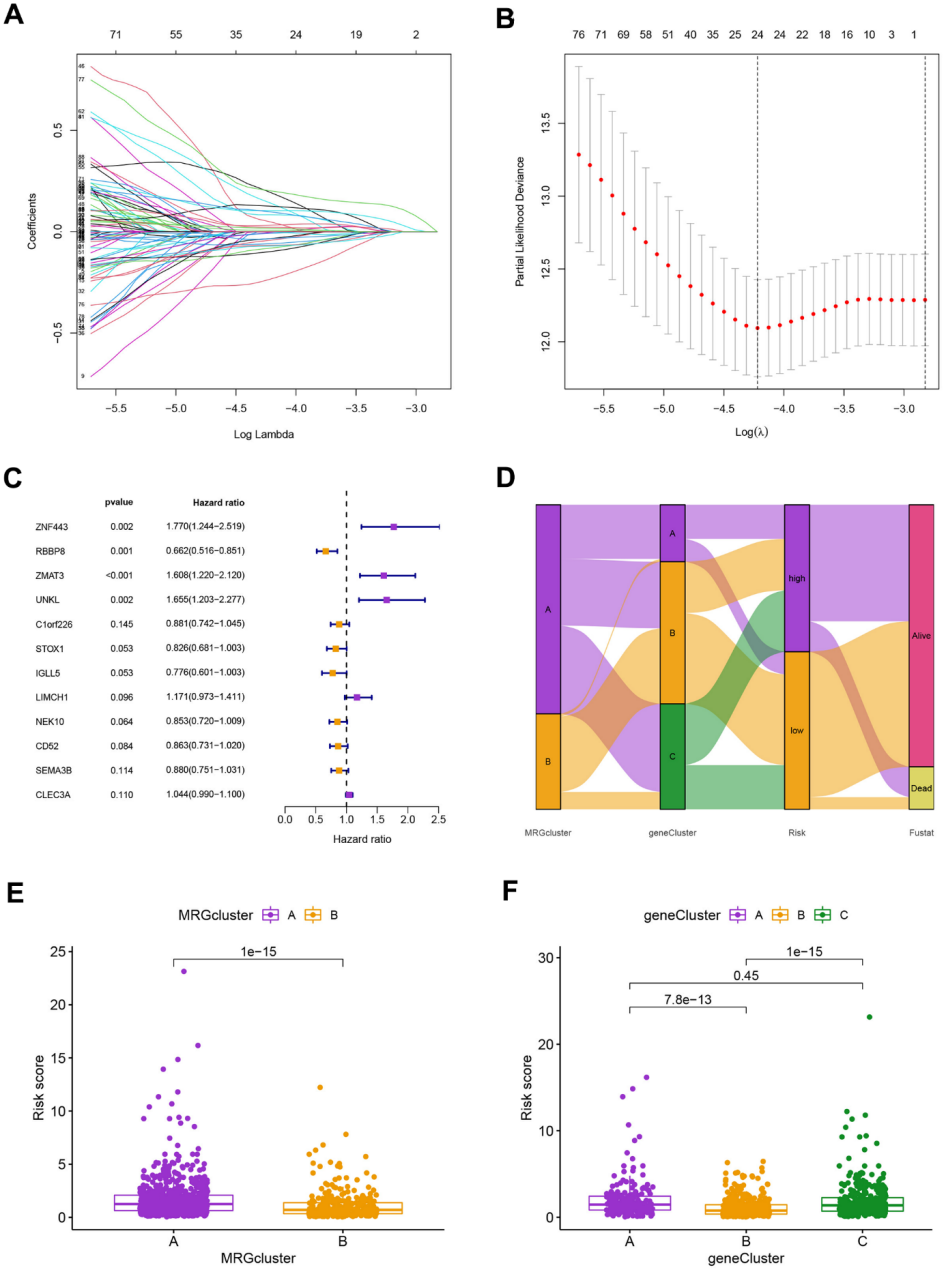
(**A**) The LASSO regression algorithm to screen candidate prognostic DEGs. (**B**) The partial likelihood deviance with changing of log(λ) in LASSO regression analysis. (**C**) The forest plot showing the relationship of prognostic-related DEGs with prognosis in BC patients. (**D**) The relationship between the MRG cluster, gene cluster, risk groups, and survival status was visualized using the Sankey diagram. (**E**, **F**) The distribution of risk scores for the two MRG clusters and three gene clusters, respectively.

Using the “caret package” in R, we first randomly divided the patients into training and testing groups at a ratio of 0.7:0.3 to examine the independent prognostic significance of the MRGs for BC patients. Next, LASSO and univariate Cox regression analysis were carried out (Figure 6A–6C). LASSO Cox regression analysis using the “glmnet” software to find the MRGs with the best prognostic value (Figure 6A, 6B). As shown in Figure 6C, 12 genes associated with the OS of BC patients were initially screened using univariate Cox proportional hazard regression analysis. The relationship between the MRG cluster, gene cluster, risk groups, and survival status was depicted using a Sankey diagram (Figure 6D). The risk score distributions for the two MRG clusters and three gene clusters are shown in Figure 6E, 6F.

**Figure 7:**
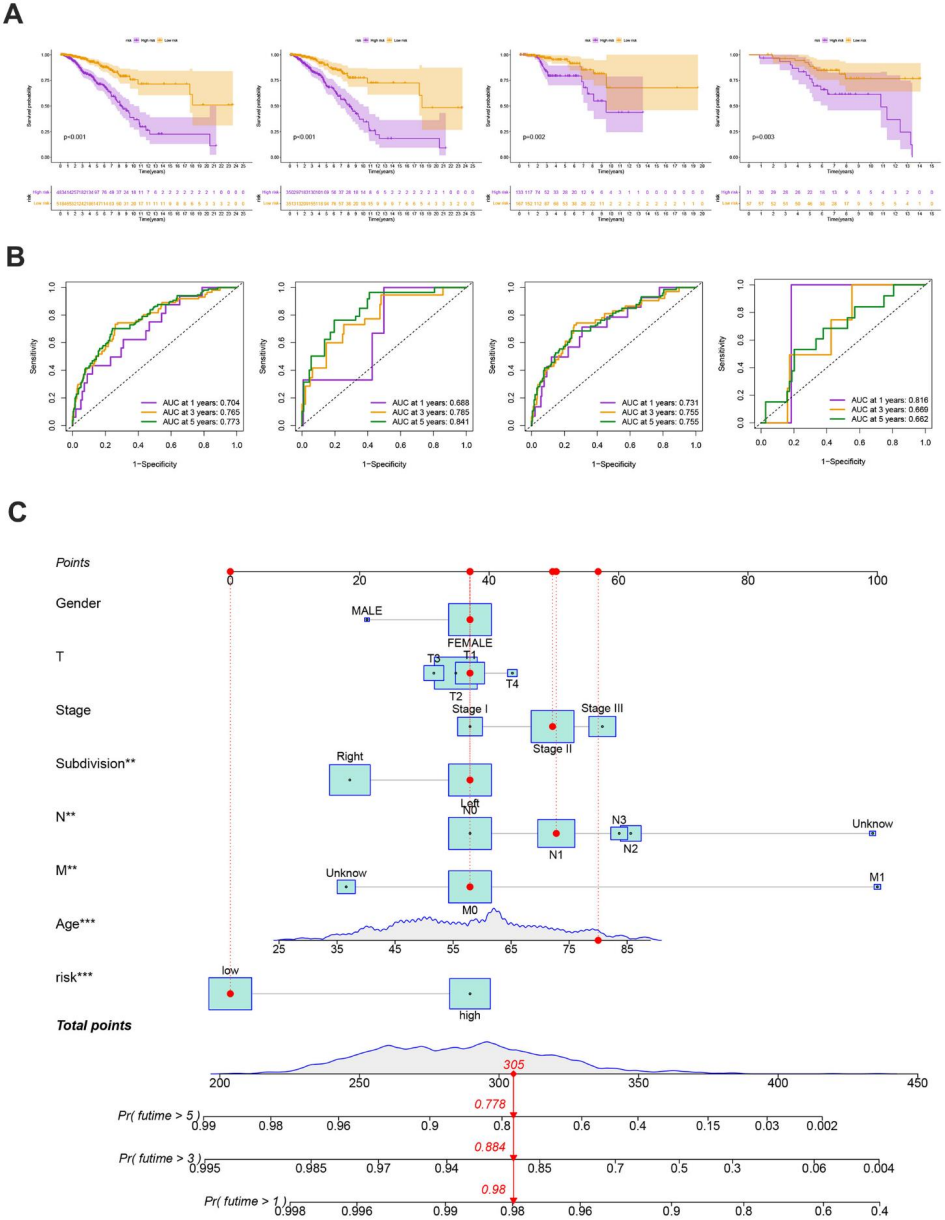
(**A**) The Kaplan-Meier analysis of high and low risk group in the entire cohort, training and testing sets, respectively. P-values calculated by log-rank test. (**B**) The ROC curves for the 1-, 3-, and 5-year AUC values in the entire cohort, training, and testing sets, respectively. (**C**) Nomogram based on risk score and clinical variables was reliable and sensitive for predicting survival in patients with BC.

Our findings supported the use of the entire set, the training set, the testing set, and the GEO external validation set. Figure 7A’s Kaplan-Meier analysis indicates that patients in the low-risk group will likely survive longer. The ROC curves showed the model’s high sensitivity and specificity for predicting survival, and the entire set’s 5-year AUC value was 0.773 (Figure 7B). Figure 7A, 7B shows the analysis for the training set, the testing set, and the GEO external validation set, which demonstrates the model’s dependability. The nomogram containing the model and clinical features was performed accurately and sensitively for predicting survival in BC patients (Figure 7C).

### Evaluation of TME

**Figure 8:**
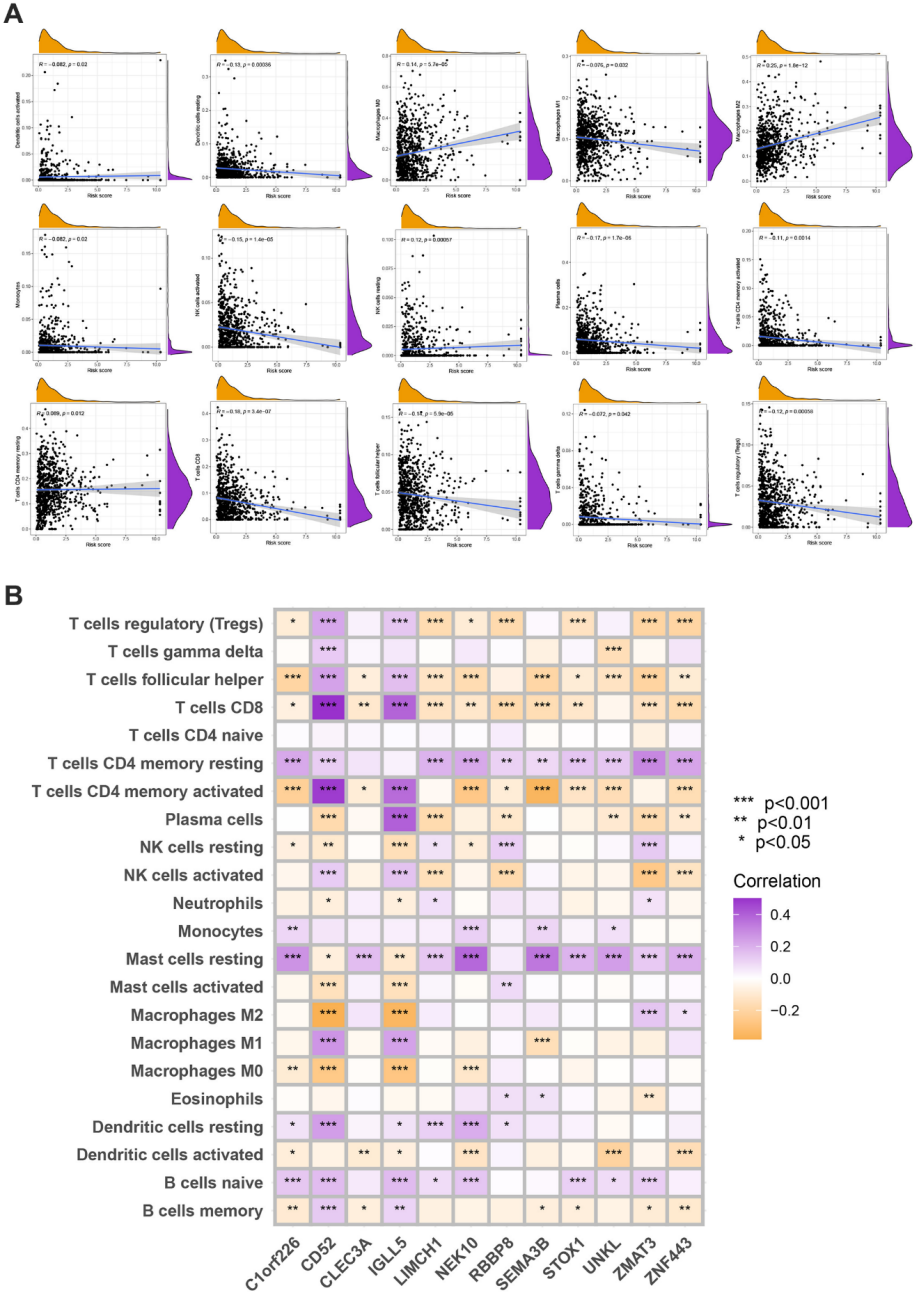
(**A**) Spearman’s correlation coefficients were computed to investigate the potential relationship between risk score and immune cell infiltration status. (**B**) Spearman’s correlation coefficients were computed to investigate the potential relationship between immune cells infiltration levels and gene expression levels. Colors from orange to purple indicate the trend of correlation from negative to positive. *P<0.05, **P<0.01, ***P<0.001.

The following data was evaluated using the CIBERSORT algorithm. The scatter diagrams revealed that the risk score was inversely correlated with resting dendritic cells, M1 macrophages, monocytes, plasma cells, activated NK cells, follicular helper T cells, gamma delta T cells, regulatory T cells, and activated memory CD4 + T cells (Figure 8A). We also explore the relation between the number of immune cells and 12 genes in this model and found a significant correlation between most immune cells and 12 genes (Figure 8B).

### Mutation, immunotherapy response, and drug susceptibility analysis

**Figure 9:**
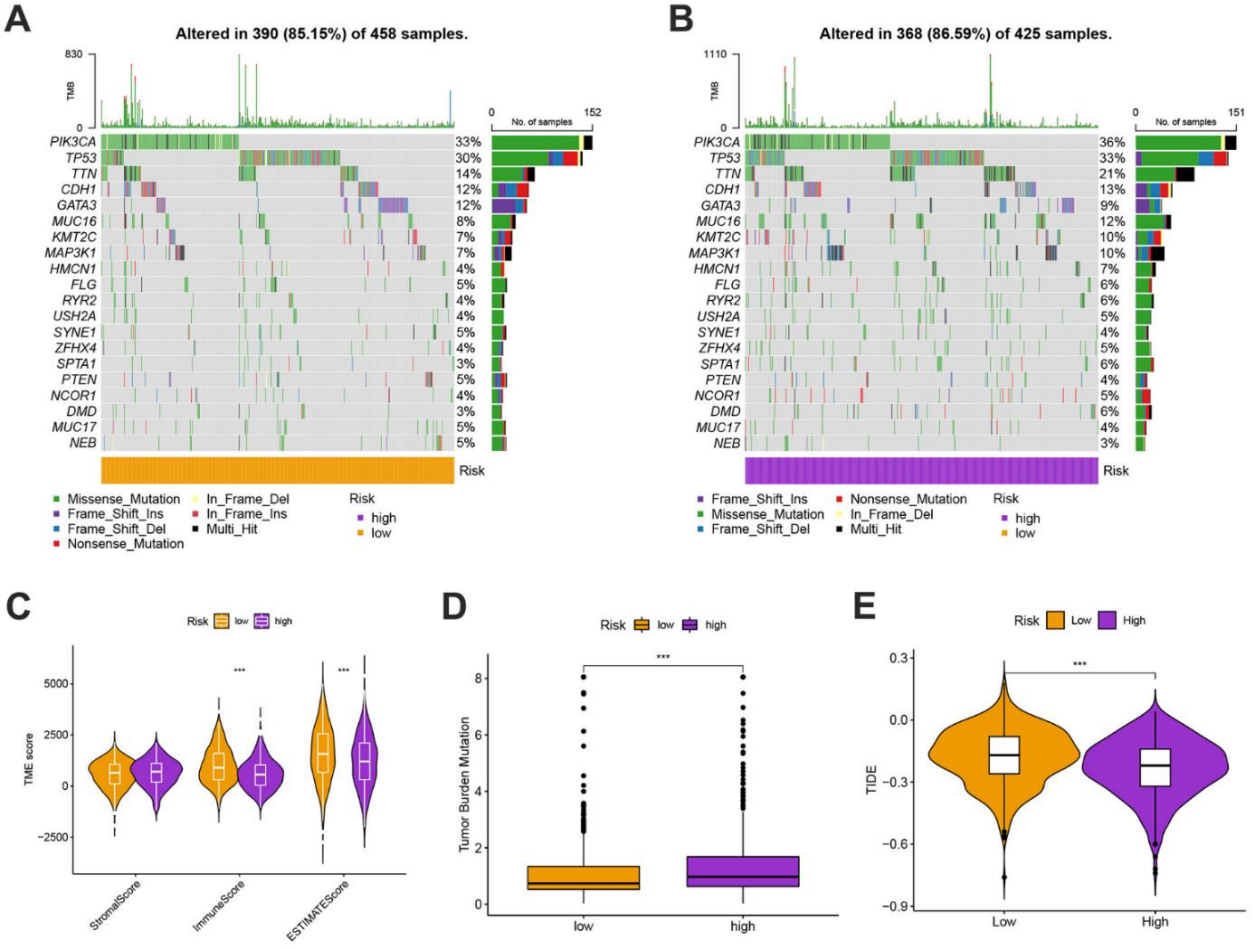
(**A**, **B**) The genes with top 10 mutations frequency in the high- and low-risk groups including PIK3CA, TP53, TTN, CDH1, GATA3, MUC16, KMT2C, MAP3K1, HMCN1, and FLG. (**C**) The ESITIMATE analysis showing that the high-risk scores were linked to a low stromal score while the low-risk scores were highly correlated with a high immune score. (**D**) The comparison of TMB scores in high and low risk groups. (**E**) TIDE scores were lower in the high-risk group, suggesting that the high risk score was more responsive to immunotherapy.

**Figure 10:**
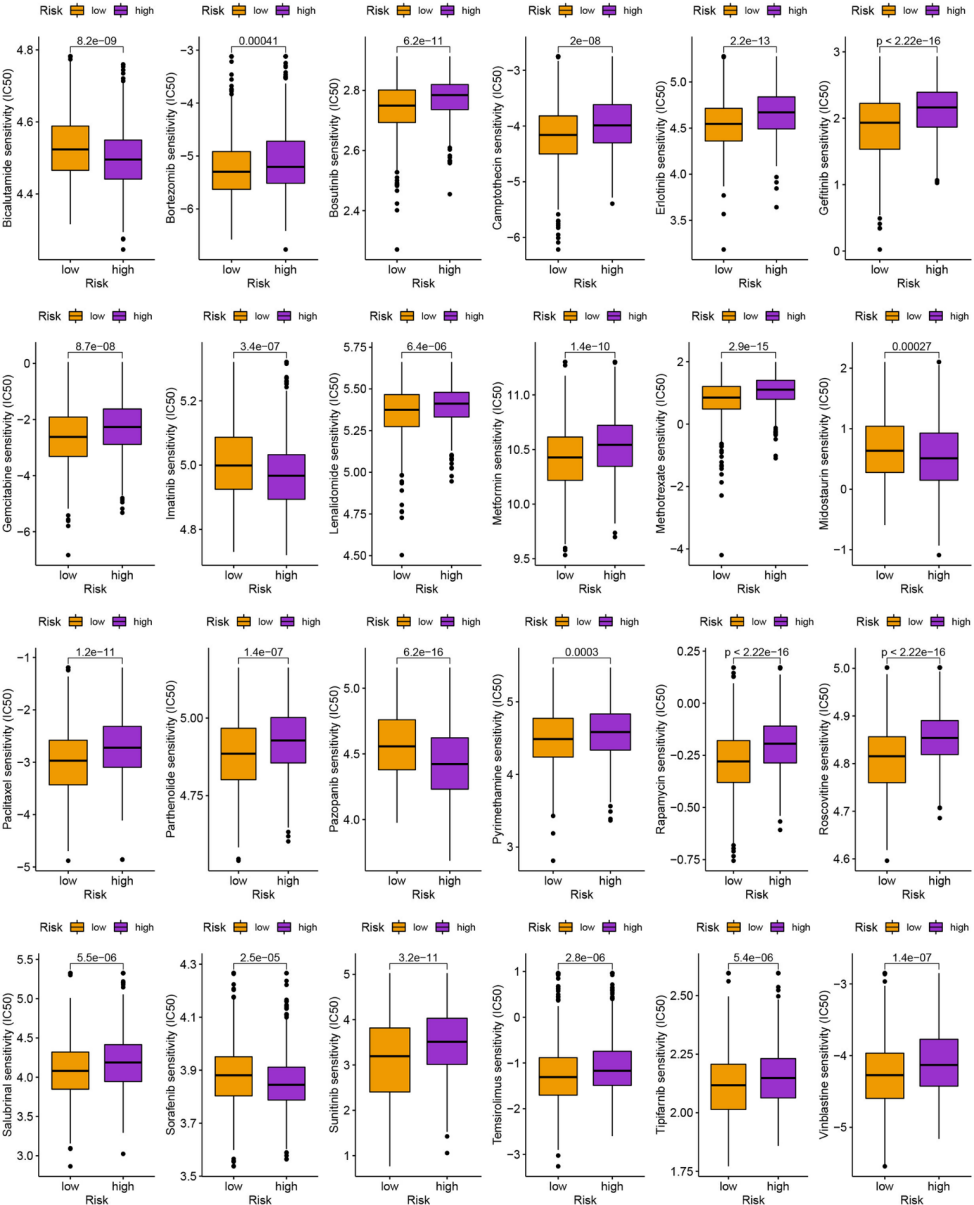
The comparison of IC50 value of anti-tumor drugs in high- and low-risk group.

We next looked at how the distribution of somatic mutations varied in the TCGA-BC cohort. In the high- and low-risk groups, the top 10 mutations were PIK3CA, TP53, TTN, CDH1, GATA3, MUC16, KMT2C, MAP3K1, HMCN1, and FLG (Figure 9A, 9B). When compared to patients in low-risk score group, those in high-risk score group exhibited greater frequencies of PIK3CA, TP53, TTN, and CDH1 mutations. Compared to the low-risk group, patients in the high-risk group had an obviously higher frequency of PIK3CA mutations than those in the low-risk group. High-risk groups were related to a high stromal score, while low-risk groups were associated with higher immune scores (Figure 9C). Furthermore, low OS was linked to increased TBM (p<0.001; Figure 9D). The high-risk group’s TIDE score was lower, indicating that they may have been more responsive to immunotherapy (Figure 9E). Furthermore, we observed significant differences in treatment susceptibility between the two patient groups by comparing the IC50 of commonly used cancer drugs (Figure 10).

### Analysis of drug susceptibility, mutation, and immunotherapy response

**Figure 11:**
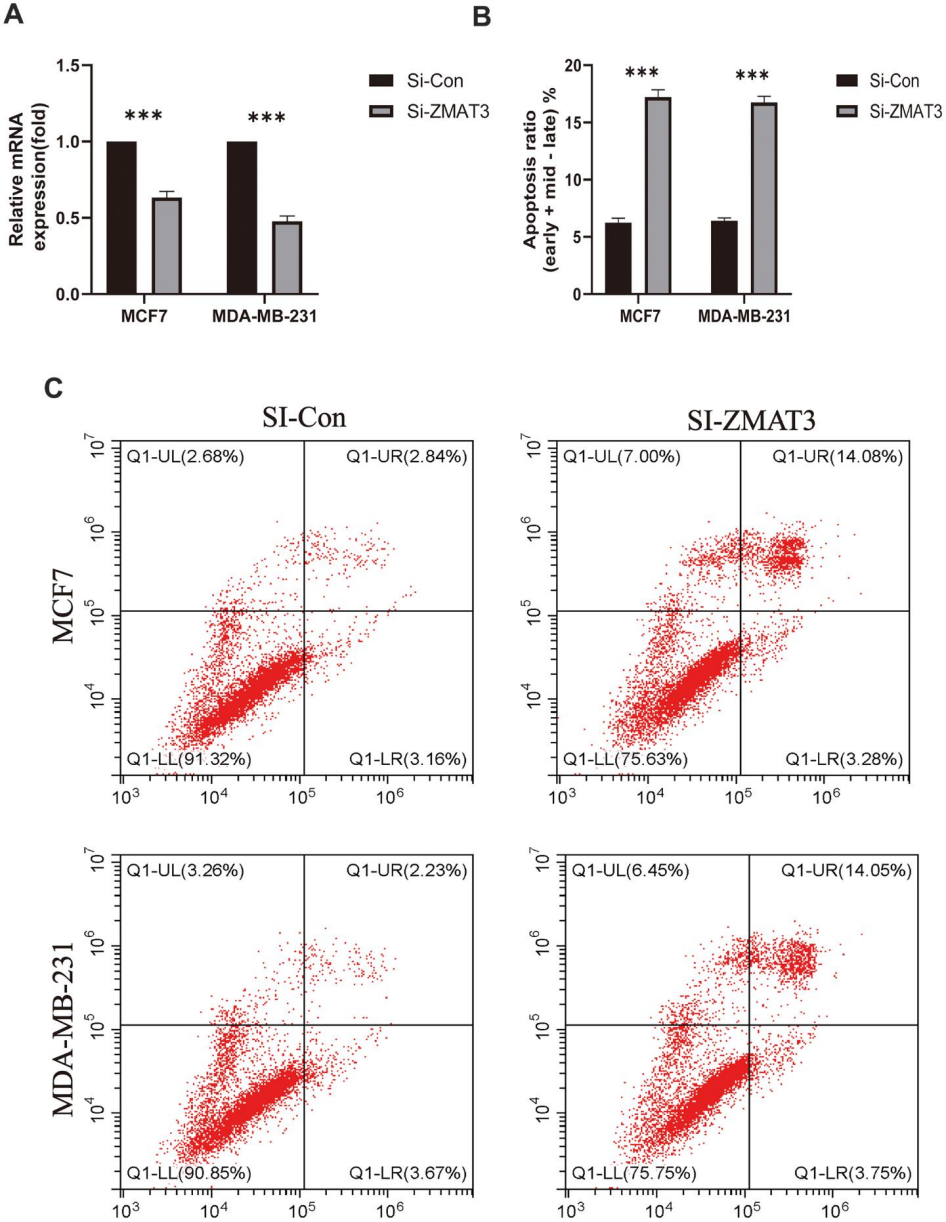
(**A**) qPCR was used to detect ZMAT3 expression. (**B**) Apoptosis ratio (early/late) % of each group. (**C**) Results of flow cytometry for each group.

To further examine the clinical impact of target genes, the ZMAT3 gene was knocked down for subsequent analysis. Following transfection with siRNA, a reduction in ZMAT3 mRNA expression was observed in both MDA-MB-231 and MCF-7 cells. Knockdown of ZMAT3 induced apoptosis in the two cell lines, as reflected in the flow cytometry results (Figure 11B, 11C), with higher numbers of early and late apoptotic cells observed compared to the control group (Figure 11A). These findings suggest that ZMAT3 may play a role in the regulation of apoptosis in BC cells and could be a potential therapeutic target.

### The oncogenic trait of ZMAT3 in breast cancer

**Figure 12:**
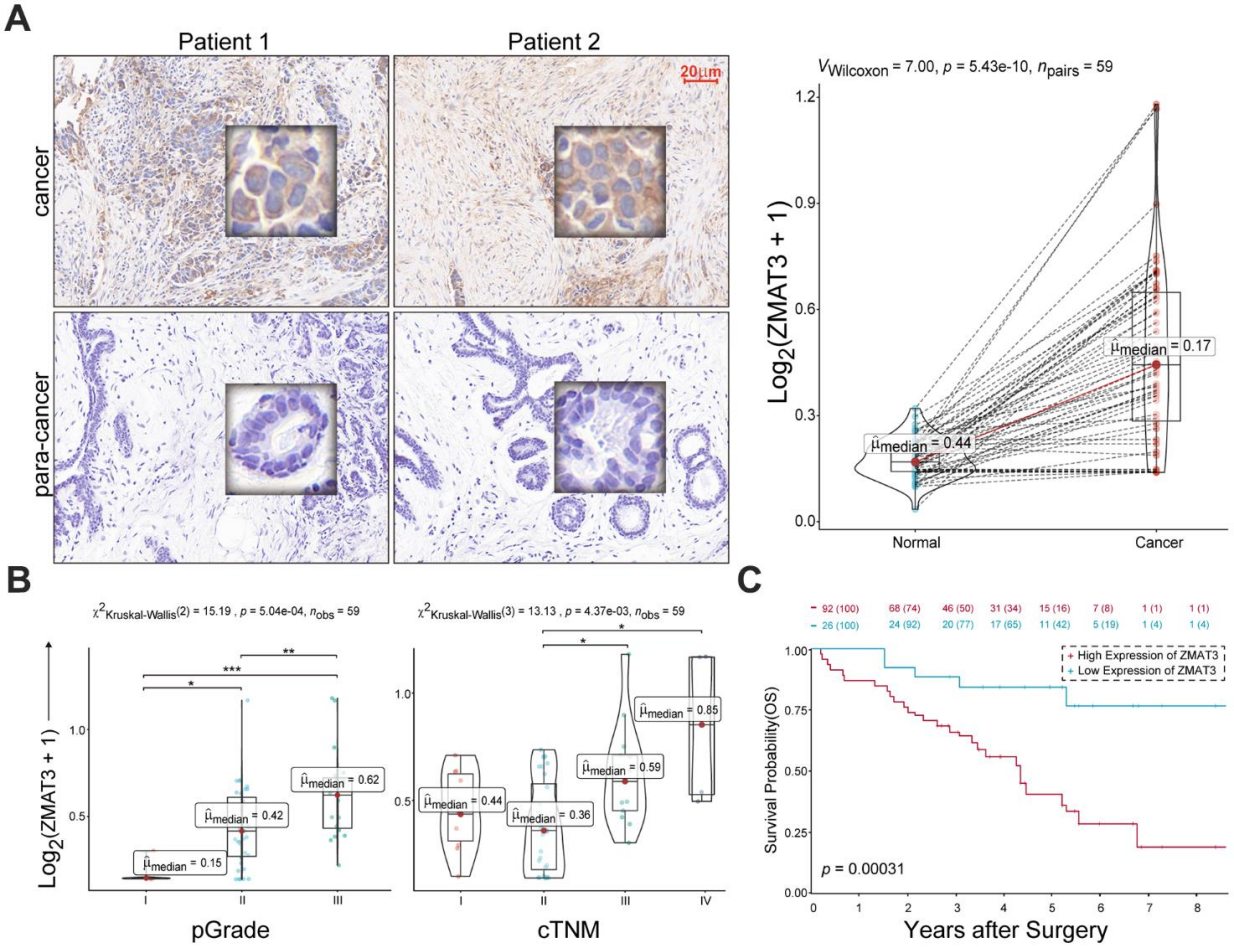
(**A**) Left: representative IHC images of ZMAT3 in breast cancer and the corresponding para-cancerous mammary duct. Scale bar = 20 μm. Right: the MOD values for the staining scores. (**B**) The correlations of MOD value of ZMAT3 with pathological grade (left) and clinical TNM stage (right), respectively. (**C**) Kaplan-Meier survival analysis for breast cancer patients with higher and lower expression levels of ZMAT3.

We identified the significant increase of ZMAT3 expression in cancer tissues compared to the para-cancerous mammary duct (Figure 12A). The MOD values of ZMAT3 that were also identified presented positive correlations with pathological grade (Figure 12B, left) and clinical TNM stage (Figure 12B, right), which suggested ZMAT3 may function as the critical oncogene in breast cancer progression. Moreover, the survival analysis demonstrated that high expression of ZMAT3 predicted a poor prognosis (Figure 12C).

Collectively, these findings indicate that the up-regulated ZMAT3 could be favourable for the malignant phenotypes of breast cancer.

## DISCUSSION

Methylation, which involves significant modifications of proteins and nucleic acids, has been strongly associated with cancer [17]. Methylation, similar to necrosis, plays a crucial role in regulating cancer progression, metastasis, and spread as an important cellular response. RNA methylation can also have an impact on the tumor immunological microenvironment and the prognosis of patients [25–28]. For example, In BC cells, hypoxia-inducible factor-1α and 2α dependent ALKBH5 can be activated, which results in NANOG mRNA being demethylated and influences the formation and incidence of tumors [29]. when the m^6^A methyltransferase is silenced, the stability of gene expression is impacted by the inhibition of the TP53 signaling pathway (a critical tumor suppressor gene) [30]. Similarly, m^1^A was reported to improve the translation efficiency and involved in determining the mRNA stability of BC [31]. DNMT3B and ALYREF (m5C regulators) were upregulated in BC and their high expression hint unfavorable prognosis [32]. The decreased levels of m6A (methylation) on ADAM19 in glioblastoma multiforme result in increased expression, which enhances the development and self-renewal of glioblastoma stem-like cells. This, in turn, ultimately leads to carcinogenesis in glioblastoma. Reduced m^6^A levels result in AKT activation, increased proliferation, and tumorigenicity in endometrial cancer cells in about 70% of endometrial cancers. rectal cancer patients with different consensus clusters of m^6^A RNA methylation regulators showed a significant difference in overall survival rate [27, 33]. Muraoka et al. demonstrated the viability of employing miR-34b/c methylation as a diagnosis and prognosis for malignant pleural mesothelioma in the context of lung cancer. Additionally, Lian et al. reported that RNA 5hmC methylation serves as a melanoma prognostic marker [34]. Five m^6^A RNA methylation regulatory factors, including HNRNPA2B1, HNRNPC, KIAA1429/VIRMA, RBM15, and METTL3, have been linked to lung cancer patients’ overall survival rates, according to a different study. Actually, as many genes affected by RNA methylation, including BCL2, FOXM1, HBXIP, and SOCS2, are crucial in regulating tumor development, RNA methylation is not only associated with carcinogenesis in diverse forms of cancer [35–37], Additionally, it is essential for tumor suppression since some loci’s lack of RNA methylation results in carcinogenesis [38]. According to a substantial number of studies, the overall effects and characteristics of different MRGs (methylated RNA genes) on the adjustment of tumor microenvironment (TME) infiltration have not been fully elucidated yet. However, the majority of research has concentrated on a single MRG or TME cell. According to recent research, MRG is highly aberrant at the genomic and transcriptional levels in BC [39, 40].

We distinguished two distinct molecular subtypes based on 57 MRGs. Those with MRG cluster A of OS have more severe OS than patients with MRG cluster B, and the two clusters of OS have quite different natures.

Changes in mRNA transcriptomes across different methylation subtypes are significantly related to biological pathways involving MRG and the immune system. We discovered three genetic clusters using the DEGs in the two MRG clusters. According to the data, MRG may be used to predict clinical outcomes and the effectiveness of immunotherapy in BC. We were able to demonstrate their prognostic abilities as a result and identify the trustworthy prognostic risk score. Higher and lower risk scores were seen in immune-activated and inhibition-driven MRG clusters, respectively. The TME, prognosis, mutation, and therapy susceptibility among various patient populations varied significantly. Then, utilizing a fusion of risk ratings and tumor stage, we produced a quantitative Normandy map that dramatically enhanced performance and made it simpler to apply risk scores. This prognostic model can be used to categorize BC patients, aid in a better understanding of the molecular causes of BC, and offer novel ideas for focused treatment. Our study, leveraging the CIBERSORT deconvolution method, has unveiled significant disparities in immune cell infiltration across distinct MRG groups in breast cancer, highlighting the complexity of immune landscapes within subtypes. The observed dominance of specific immune cells, such as cytotoxic T cells or regulatory T cells in certain groups, points to varying degrees of tumor aggressiveness or immunosuppression, critical for tailoring prognosis and immunotherapy approaches. Notably, a pronounced presence of cytotoxic T cells could indicate a favorable response to immune checkpoint inhibitors, steering therapeutic strategies. These patterns of differential immune infiltration might also mirror the spectrum of immune evasion mechanisms, a reflection of the tumor’s adaptive pressures on the immune system. Unraveling these mechanisms is pivotal for crafting bespoke immunotherapies aligned with the unique immune profiles of MRG-defined subgroups. The distinct immune infiltration landscapes underscore the integral role of immunogenomic analyses in oncology, enriching our understanding of tumor-immune dynamics and opening new avenues for personalized immunotherapeutic interventions, necessitating further exploratory studies to elucidate the functional implications of these immune cell infiltration differences.

After receiving standard therapy, BC patients have a poor prognosis because of elevated checkpoints, tumor-infiltrating cells, and neoantigens [41, 42]. The fact that patients with BC still have a heterogeneous prognosis despite recent therapeutic advancements emphasizes the crucial part TME plays in the development and progression of BC tumors [43]. Immune cells such as macrophages, lymphocytes, and granulocytes are among the key biological components of TME [44]. To aid in survival, these cells take part in a range of immunological reactions and actions, such as the coordination of inflammatory responses by tumors [45, 46]. Additional data point to a critical function for TME in cancer development, progression, and therapy resistance [47]. In the present investigation, immune activation-driven MRG (cluster B) was linked with lower risk scores than immunological inhibition-driven MRG (cluster A). In terms of the TME characteristics and relative richness of 22 immune cells, we discovered that these two molecular clusters and various risk scores differed significantly from one another.

These findings suggest that MRG plays a crucial role in the growth of BC. An increasing body of evidence supports the significance of potent T cells, memory T cells, and T cells in the immunological response against BC. CD4+ T cells play a significant role in tumor management, as they can either enhance or suppress anti-tumor responses. However, CD8+ T cells are generally considered to be the key drivers of anti-tumor immunity. They are believed to be primarily responsible for exerting anti-tumor effects and facilitating immune responses against cancer cells. For instance, by stimulating a variety of innate immune cell types, including CD8+ T cells, NK cells, and others, conventional CD4+ T cells can aid in the control of tumors [48]. Since tumor-infiltrating T cell concentrations in BC samples were higher than those in healthy tissues, this suggested a favorable prognosis. Increased infiltration of activated memory CD4+ and CD8+ T cells and T cells indicated that B cluster and low-risk groups had better prognoses and likely contributed to the advancement of BC. The importance of effector T cell, memory T cell, and T cell differentiation is becoming increasingly clear [25]. M1 macrophages and M2 macrophages are the two phenotypes of tumor-associated macrophages (as we all known, the former prevents the spread of cancer, and the latter promotes cancer progression). According to this study, patients with low-risk scores may benefit from immunotherapy because macrophages M1 was more abundant in the groups with low-risk scores. Immunosuppressive M2 macrophages support matrix remodeling, which encourages the growth of malignancies [49]. As in previous research, we discovered that M1 macrophage infiltration increased in MRG cluster B groups with low risk-score and good prognosis, but not in MRG cluster A groups with high risk-score and poor prognosis.

Although, it was shown that a large number of studies found that B cells support the immunological response. In Hodgkin lymphoma, for instance, studies have indicated that B cell enrichment is positively correlated with responsiveness to PD-1 inhibition, as described by Vari et al. This suggests that B cells may contribute to the effectiveness of immune checkpoint blockade therapies in certain cancer types [50]. According to Engelhard V et al., patients who reacted to immune checkpoint inhibition had significantly higher levels of the B cell-related genes than those who did not [51]. According to Hollern et al. immune checkpoint therapy induces T follicular helper cell activation of B cells to facilitate the anti-tumor response in these models [52]. Additionally, a favorable prognosis in BC was associated with tumor-infiltrating B cells. Numerous studies have demonstrated that T cells and B cells interact and go through cooperative selection, specialization, and clonal growth in tertiary lymphoid structures associated with tumors. Importantly, B cells can interact with T cells by presenting them with homologous tumor-derived antigens; the functional outcomes of such interactions are influenced by the B cell phenotype [53]. The findings of this study showed that B cells represent a unique immunotherapy target and may be an effective cancer-fighting tool, rather than merely incidental contributions to anti-cancer immunotherapy. This is consistent with our findings.

In this study, the expression levels of a part of immune cells were found to be different obviously in the risk model of MRGs. Our study revealed that the risk score was inversely correlated with resting dendritic cells, M1 macrophages, monocytes, plasma cells, active NK cells, follicular helper T cells, gamma delta T cells, regulatory T cells, and activated memory CD4 + T cells. This implies that BC immune cell infiltration is related to the risk model created using MRGs. Our study shows a significant correlation between most immune cells and 12 genes, The high-risk score group was related to a high stromal score, and the low-risk score group was closely associated with a high immune score. Yan-Fei Ma et al. demonstrated Early breast cancer was shown to have high levels of CD52 expression, and these levels were correlated with a good prognosis [54]. Higher infiltrations of M1 macrophages, monocytes, T follicular helper cells, and resting memory CD4 T cells were caused by overexpression of CD52. Downregulation of CD52 led to significant M2 macrophage infiltration [54]. Studies have shown that some TME and IGLL5 expression are linked. Additionally, three different types of TME are positively linked with IGLL5 expression, according to our immune infiltration findings. Potential for IGLL5 as a predictive biomarker of clear cell renal cell carcinoma [55]. According to Scannell Bryan et al., the NEK10 variant may play a role in the incidence of breast cancer [56]. Our study established a risk model for BC and found evidence of MRGs involvement; however, further clinical BC tissue samples and cell tests are required to corroborate this. A high-risk score is an independent risk factor for a poor prognosis in BC patients and is associated with patient outcomes.

The study has several limitations. First, all the analysis is based entirely on data from a common database. Therefore, an inherent bias in case selection may have influenced the results. Second, further prospective studies and additional *in vitro* and *in vivo* studies are needed to validate our findings. In addition, most datasets were unable to analyze data on important clinical variables (such as surgery, neoadjuvant chemotherapy, and chemoradiotherapy) that could affect the outcome of immune response and methylation status.

## CONCLUSIONS

Through our comprehensive investigation of MRGs, we have discovered their influence on the tumor microenvironment (TME), clinicopathological characteristics, and various prognostic regulatory systems. Furthermore, we have identified the therapeutic roles of immunotherapy and commonly used anti-tumor medications. These findings emphasize the clinical significance of MRGs and provide novel insights to guide both immunotherapy and conventional anti-tumor strategies in BC patients.

## Supplementary Material

Supplementary Table 1

Supplementary Table 2

Supplementary Table 3

Supplementary Table 4

Supplementary Table 5

Supplementary File 1
