# Supplementary material for: Breast cancer clinical outcomes and tumor immune microenvironment: cross-dialogue of multiple epigenetic modification profiles
Source: Aging (Albany NY). 2024 May 22;16(10):8998–9022. doi: 10.18632/aging.205853 (PMC11164499; doi:10.18632/aging.205853)
Supplement: Supplementary File 1 [file aging-16-205853-s006.docx]

**Supplementary File 1. The R code script.**

###########Figure 1

#

library(ggplot2)

library(ggpubr)

library(tidyverse)

mtcars=read.table("clipboard",sep = "\t",header = T,check.names = F)

symnum.args <- list(cutpoints = c(0, 0.001, 0.01, 0.05, 1),

symbols = c("***", "**", "*", "ns"))

ggplot(mtcars,aes(x = factor(Group),y = RSPO2)) +

geom_violin(aes(fill = factor(Group)),color="black",

width = 0.7,size=0.4)+

geom_boxplot(aes(fill = factor(Group)),

width = 0.2,

notch = F,

notchwidth = 0.5,

outlier.color = 'black',

size = 0.2) +

geom_jitter(color="black",

size = 3,alpha = 0.5,

position = position_jitter(width = 0.2)) +

theme_classic(base_size = 18) +

theme_classic(base_size = 18) +

theme(aspect.ratio = 1.5,

axis.text.x = element_text(angle = 45,hjust = 1,color = 'black',size = 16),

legend.position = 'none') +

scale_colour_manual(values = c("#4DAF4A","#E41A1C"))+scale_fill_manual(values = c("#4DAF4A","#E41A1C"))+

xlab('')+ggtitle("TCGA")+theme(plot.title = element_text(hjust = 0.5))+

stat_compare_means(comparisons = list(c('Control','ESCA')),

size = 5,

step.increase = 0.13,

symnum.args =symnum.args)

#

cluster=read.table("clipboard",sep = "\t",header = T,check.names = F)

cluster$Pathologic_stage=factor(cluster$Pathologic_stage, levels=c("Stage I","Stage II","Stage III","Stage IV"))

my_comparisons=list(c("Stage I","Stage II"),c("Stage I","Stage III"),c("Stage I","Stage IV"),c("Stage II","Stage III"),c("Stage II","Stage IV"),c("Stage III","Stage IV"))

pdf(file="Pathologic_stage-RSPO2.pdf",width=6,height=6) #"#337AB7","#D9534F","#5CB85C","#F0AD4E"

ggviolin(cluster, x="Pathologic_stage", y="RSPO2", fill = "Pathologic_stage",color = "black",width = 0.5,

palette = c("#337AB7","#D9534F","#5CB85C","#F0AD4E"),ylab=c("Expression of RSPO2"),bxp.errorbar=T,add = "boxplot")+

stat_compare_means(comparisons = my_comparisons, method="t.test",symnum.args=list(cutpoints = c(0, 0.001, 0.01, 0.05, 1), symbols = c("***", "**", "*", "ns")), label = "p.signif") #默认method是wilcoxon检验，看情况修改

dev.off()

##########Figure 2

#

library(ggplot2)

library(ggcor)

siglec15 <- read.table("RSPO2 exp.txt", row.names = 1, check.names = F,header = T,sep = "\t")

immPath.score <- read.table("immune process.txt", check.names = F,row.names = 1,header = T)

immCorSiglec15 <- NULL

for (i in rownames(immPath.score)) {

cr <- cor.test(as.numeric(immPath.score[i,]),

as.numeric(siglec15),

method = "pearson")

immCorSiglec15 <- rbind.data.frame(immCorSiglec15,

data.frame(gene = "RSPO2",

path = i,

r = cr$estimate,

p = cr$p.value,

stringsAsFactors = F),

stringsAsFactors = F)

}

immCorSiglec15$sign <- ifelse(immCorSiglec15$r > 0,"pos","neg")

immCorSiglec15$absR <- abs(immCorSiglec15$r)

immCorSiglec15$rSeg <- as.character(cut(immCorSiglec15$absR,c(0,0.25,0.5,0.75,1),labels = c("0.25","0.50","0.75","1.00"),include.lowest = T))

immCorSiglec15$pSeg <- as.character(cut(immCorSiglec15$p,c(0,0.001,0.01,0.05,1),labels = c("<0.001","<0.01","<0.05","ns"),include.lowest = T))

immCorSiglec15$rSeg <- factor(immCorSiglec15$rSeg, levels = c("0.25","0.50","0.75","1.00"))

immCorSiglec15$pSeg <- factor(immCorSiglec15$pSeg, levels = c("<0.001","<0.01","<0.05","Not Applicable","ns"))

immCorSiglec15$sign <- factor(immCorSiglec15$sign, levels = c("pos","neg"))

p1 <- quickcor(t(immPath.score),

type = "lower",

show.diag = TRUE) +

geom_colour() + #geom_circle2()或者geom_square()

anno_link(data = immCorSiglec15,

mapping = aes(colour = pSeg, size = rSeg, linetype = sign),

spec.key = "gene",

env.key = "path",

diag.label = FALSE) +

scale_size_manual(values = c(0.5, 1, 1.5, 2)) +

scale_color_manual(values = c("#19A078","#DA6003","#7570B4","#E8288E","#65A818")) +

scale_fill_gradient2(low = "#9483E1",mid = "white",high = "#80B1D3",midpoint=0) +

remove_axis("x")

p1

ggsave(filename = "ggcor-immune process.pdf", width = 12,height = 10)

#

library(ggplot2)

library(ggpubr)

clinical<-read.table("clipboard",header = T,sep = "\t",stringsAsFactors = F,check.names = F)

clinical$Group<-factor(clinical$Group,levels = c("Low expression","High expression"))

fit<-cor.test(clinical$RSPO2,clinical$ESTIMATEScore)#pearson相关

fig11a<-ggplot(clinical,aes(x = RSPO2,y = ESTIMATEScore ,colour = Group))+

geom_point(size=2,alpha=1)+

geom_smooth(method = "lm", se=FALSE,color="#E1776C", formula = y ~ x)+

theme_bw()+

theme(legend.position = "none",

axis.title = element_text(size = 14),

axis.text = element_text(size = 12,colour = "black"),

axis.ticks = element_line(size = 1,colour = "black"))+

annotate("text", x=0, y=0.4, label=paste0("R = ",signif(fit$estimate,2),"\n",

"P = ",signif(fit$p.value,1)),size = 6) +

scale_color_manual(values =c("#377EB8","#4DAF4A"))

fig11a_top<-ggplot(clinical,aes(x = RSPO2,y = Group,fill = Group))+

geom_boxplot()+

theme_bw()+

theme(panel.background = element_blank(),

panel.border = element_blank(),

panel.grid = element_blank(),

axis.title = element_blank(),

axis.text = element_blank(),

axis.ticks = element_blank(),

legend.position = "top")+

scale_fill_manual(values =c("#377EB8","#4DAF4A"))

my_compare<-list(c("Low expression","High expression"))

fig11a_right<-ggboxplot(clinical, x="Group", y="ESTIMATEScore",scales = "free_x",fill = "Group",

short.panel.labs = F,outlier.shape = NA)+

theme_bw()+

theme(panel.background = element_blank(),

panel.border = element_blank(),

panel.grid = element_blank(),

axis.title = element_blank(),

axis.text = element_blank(),

axis.ticks = element_blank(),

legend.position = "none")+

scale_fill_manual(values =c("#377EB8","#4DAF4A"))+

stat_compare_means(comparisons = my_compare,method="wilcox.test",label = "p.signif",

tip.length=0)

empty <- ggplot()+geom_point(aes(1,1), colour="white") +

theme(

plot.background = element_blank(),

panel.grid.major = element_blank(),

panel.grid.minor = element_blank(),

panel.border = element_blank(),

panel.background = element_blank(),

axis.title.x = element_blank(),

axis.title.y = element_blank(),

axis.text.x = element_blank(),

axis.text.y = element_blank(),

axis.ticks = element_blank()

,plot.margin=unit(c(0.1, 0.1, 0, 0), "inches")

)

pg1=ggpubr::ggarrange(fig11a_top,fig11a, ncol = 1, nrow = 2,heights = c(0.3,1),align = "v")

pg2=ggpubr::ggarrange(empty,fig11a_right, ncol = 1, nrow = 2,heights = c(0.3,1),align = "v")

fig11a=ggpubr::ggarrange(pg1,pg2, ncol = 2, nrow = 1,widths = c(1,0.3),align = "h")

fig11a

#

library(ggpubr)

Type=read.table("RSPO2 exp.txt",sep="\t",check.names=F,row.names=1,header=T)

rt=read.table("HLA.txt",sep="\t",check.names=F,row.names=1,header=T)

Type=Type[colnames(rt),]

rt=t(rt)

data=data.frame()

for(i in colnames(rt)){

data=rbind(data,cbind(expression=(rt[,i]),gene=i,Group=as.vector(Type[,2])))

}

write.table(data,file="data.txt",sep="\t",quote=F)

cluster=read.table("data.txt",sep = "\t",header = T,check.names = F)

cluster$Group=factor(cluster$Group, levels=c("Low expression","High expression"))

p=ggboxplot(cluster, x="gene", y="expression", fill = "Group",color = "black",

ylab="Expression",

xlab="",

palette = c("#377EB8","#4DAF4A") )

p=p+rotate_x_text(60)

pdf(file="HLA.pdf",width=15,height=5) #输出图片文件

p+stat_compare_means(aes(group=Group),method="wilcox.test",symnum.args=list(cutpoints = c(0, 0.001, 0.01, 0.05, 1), symbols = c("***", "**", "*", "")),label = "p.signif")

dev.off()

###########Figure 3

#

logFoldChange=1

adjustP=0.05

conNum=91

treatNum=92

library(limma)

rt=read.table("sampleExp.txt",sep="\t",header=T,check.names=F)

rt=as.matrix(rt)

rownames(rt)=rt[,1]

exp=rt[,2:ncol(rt)]

dimnames=list(rownames(exp),colnames(exp))

rt=matrix(as.numeric(as.matrix(exp)),nrow=nrow(exp),dimnames=dimnames)

rt=avereps(rt)

rt=rt[rowMeans(rt)>0,]

rt=normalizeBetweenArrays(as.matrix(rt))

modType=c(rep("low",conNum),rep("high",treatNum))

design <- model.matrix(~0+factor(modType))

colnames(design) <- c("high","low")

fit <- lmFit(rt,design)

cont.matrix<-makeContrasts(high-low,levels=design)

fit2 <- contrasts.fit(fit, cont.matrix)

fit2 <- eBayes(fit2)

allDiff=topTable(fit2,adjust='fdr',number=200000)

write.table(allDiff,file="diff-All.txt",sep="\t",quote=F)

#write table

diffSig <- allDiff[with(allDiff, (abs(logFC)>logFoldChange & adj.P.Val < adjustP )), ]

diffSigOut=rbind(id=colnames(diffSig),diffSig)

write.table(diffSigOut,file="sig Diff.xls",sep="\t",quote=F,col.names=F)

#

library(fgsea)

library(clusterProfiler)

deg.test<- read.table("diff-All.txt",sep = "\t",header = T,check.names = F,row.names = 1)

deg.test$logFC <- as.numeric(as.character(deg.test$logFC))

deg.test <- deg.test[order(deg.test$logFC, decreasing = T), ]

si.id <- deg.test$logFC

names(si.id) <- rownames(deg.test)

head(si.id)

gmtfile <- "h.all.v2022.1.Hs.symbols.gmt"

hallmark <- read.gmt(gmtfile)

hallmark$term<-gsub('HALLMARK_','',hallmark$term)

hallmark.list <- hallmark %>% split(.$term) %>% lapply( "[[", 2)

fgseaRes <- fgsea(pathways = hallmark.list,

stats = si.id,

minSize=5,

maxSize=1000)

sig<-fgseaRes[fgseaRes$padj<0.05,]

sig<-sig[order(sig$NES,decreasing = T)]

topPathwaysUp <- fgseaRes[ES > 0][head(order(pval), n=5), pathway]

topPathwaysDown <- fgseaRes[ES < 0][head(order(pval), n=5), pathway]

topPathways <- c(topPathwaysUp, rev(topPathwaysDown))

length(topPathways)

pdf(file="Hallmark fgsea.pdf", width = 8, height = 6)

plotGseaTable(hallmark.list[topPathways], si.id, fgseaRes,

gseaParam = 0.5)

dev.off()

#

library(fgsea)

library(clusterProfiler)

deg.test<- read.table("diff-All.txt",sep = "\t",header = T,check.names = F,row.names = 1)

deg.test$logFC <- as.numeric(as.character(deg.test$logFC))

deg.test <- deg.test[order(deg.test$logFC, decreasing = T), ]

si.id <- deg.test$logFC

names(si.id) <- rownames(deg.test)

head(si.id)

gmtfile <- "c2.cp.reactome.v2022.1.Hs.symbols.gmt"

hallmark <- read.gmt(gmtfile)

hallmark$term<-gsub('REACTOME_','',hallmark$term)

hallmark.list <- hallmark %>% split(.$term) %>% lapply( "[[", 2)

fgseaRes <- fgsea(pathways = hallmark.list,

stats = si.id,

minSize=5,

maxSize=1000)

sig<-fgseaRes[fgseaRes$padj<0.05,]

sig<-sig[order(sig$NES,decreasing = T)]

topPathwaysUp <- fgseaRes[ES > 0][head(order(pval), n=5), pathway]

topPathwaysDown <- fgseaRes[ES < 0][head(order(pval), n=5), pathway]

topPathways <- c(topPathwaysUp, rev(topPathwaysDown))

length(topPathways)

pdf(file="Rectome fgsea.pdf", width = 8, height = 6)

plotGseaTable(hallmark.list[topPathways], si.id, fgseaRes,

gseaParam = 0.5)

dev.off()

#

library(TCGAbiolinks)

query <- GDCquery(

project = "TCGA-ESCA",

data.category = "Simple Nucleotide Variation",

data.type = "Masked Somatic Mutation",

access = "open"

)

GDCdownload(query)

GDCprepare(query, save = T,save.filename = "TCGA-ESCA_SNP.Rdata")

library(maftools)

load(file = "F:/XIANYU/2023-05-09-As Gitto-食管癌-RSPO2/TCGA-ESCA_SNP.Rdata")

maf.coad<- data

maf <- read.maf(maf.coad)

plotmafSummary(maf = maf, rmOutlier = TRUE, addStat = 'median', dashboard = TRUE)

maf.coad$Tumor_Sample_Barcode=substr(maf.coad$Tumor_Sample_Barcode,1,12)

rt=read.table("RSPO2 exp.txt",sep = "\t",header = T,check.names = F)

mut.High=maf.coad[(maf.coad$Tumor_Sample_Barcode %in% rt$gene[rt$Group=="High expression"]),]

mut.Low=maf.coad[(maf.coad$Tumor_Sample_Barcode %in% rt$gene[rt$Group=="Low expression"]),]

maf.High <- read.maf(mut.High)

pdf(file="oncoplot-High expression.pdf", width=6.5, height=6)

oncoplot(maf=maf.High, draw_titv=F)

dev.off()

maf.Low <- read.maf(mut.Low)

pdf(file="oncoplot-Low expression.pdf", width=6.5, height=6)

oncoplot(maf=maf.Low, draw_titv=F)

dev.off()

##########Figure 4

#

library("clusterProfiler")

library("org.Hs.eg.db")

library("enrichplot")

library("ggplot2")

rt=read.table("gene.txt",sep="\t",header=T,check.names=F,stringsAsFactors = F,quote = "") #含基因名的文件

gzs=toTable(org.Hs.egSYMBOL)

rt=merge(rt,gzs,by='symbol',all.x=T)

rt=rt[is.na(rt[,"gene_id"])==F,]

gene=rt$gene_id

kk <- enrichGO(gene = gene,

OrgDb = org.Hs.eg.db,

pvalueCutoff =0.05,

qvalueCutoff = 0.05, #适当调整

ont="all",

readable =T) #readable=T可以使GO以term形式出现而不是id

write.table(kk,file="GO.txt",sep="\t",quote=F,row.names = F)

library(ggplot2)

library(tidyverse)

library(ragg)

data=read.table("clipboard",sep = "\t",header = T,check.names = F)

label_data <- data

number_of_bar <- nrow(label_data)

angle <- 90 - 360 * (label_data$id-0.5) /number_of_bar # I substract 0.5 because the letter must have the angle of the center of the bars. Not extreme right(1) or extreme left (0)

label_data$hjust <- ifelse( angle < -90, 1, 0)

label_data$angle <- ifelse(angle < -90, angle+180, angle)

base_data <- data %>%

group_by(ONTOLOGY) %>%

summarize(start=min(id), end=max(id) - 1) %>%

rowwise() %>%

mutate(title=mean(c(start, end)))

head(base_data)

grid_data <- base_data

grid_data$end <- grid_data$end[ c( nrow(grid_data), 1:nrow(grid_data)-1)] + 1

grid_data$start <- grid_data$start - 1

grid_data <- grid_data[-1,]

head(grid_data)

p <- ggplot(data, aes(x=as.factor(id), y=padj, fill=ONTOLOGY)) + # Note that id is a factor. If x is numeric, there is some space between the first bar

geom_bar(aes(x=as.factor(id), y=padj, fill=ONTOLOGY), stat="identity", alpha=0.5) +

# Add a val=100/75/50/25 lines. I do it at the beginning to make sur barplots are OVER it.

#geom_segment(data=grid_data, aes(x = end+3, y = 40, xend = start, yend = 40), colour = "grey", alpha=1, size=0.3 , inherit.aes = FALSE ) +

#geom_segment(data=grid_data, aes(x = end+3, y = 30, xend = start, yend = 30), colour = "grey", alpha=1, size=0.3 , inherit.aes = FALSE ) +

#geom_segment(data=grid_data, aes(x = end+3, y = 20, xend = start, yend = 20), colour = "grey", alpha=1, size=0.3 , inherit.aes = FALSE ) +

#geom_segment(data=grid_data, aes(x = end+3, y = 10, xend = start, yend = 10), colour = "grey", alpha=1, size=0.3 , inherit.aes = FALSE ) +

# Add text showing the value of each 100/75/50/25 lines

# annotate("text", x = rep(max(data$id),4), y = c(10,20,30, 40), label = c("10","20", "30", "40") , color="blue", size=3 , angle=0, fontface="bold", hjust=1) +

theme_minimal() +

theme(

#legend.position = "none",

axis.text = element_blank(),

axis.title = element_blank(),

panel.grid = element_blank(),

plot.margin = unit(rep(-1,4), "cm")

) +

coord_polar() +

# 添加标签注释信息

geom_text(data=label_data, aes(x=id, y=padj+4, label=Description, hjust=hjust), color="black", fontface="bold",alpha=0.6, size=3, angle= label_data$angle, inherit.aes = FALSE ) +

geom_text(data=label_data, aes(x=id, y=padj-2, label=Count, hjust=hjust), color="black", fontface="bold",alpha=0.6, size=3, angle= label_data$angle, inherit.aes = FALSE ) +

# Add base line information

# 添加下划线

geom_segment(data=base_data, aes(x = start, y = -5, xend = end, yend = -5), colour = "black", alpha=0.8, size=0.8 , inherit.aes = FALSE ) +

# 添加各组的名字

geom_text(data=base_data, aes(x = title, y = -12, label=ONTOLOGY), hjust=c(1,0,0), colour = "black", alpha=0.8, size=3, fontface="bold", inherit.aes = FALSE) +

# 更改颜色

scale_fill_brewer(palette = "Set2")

pdf(file="GO-circular barplot.pdf",width=13,height=13)

p

dev.off()

#

kk <- enrichKEGG(gene = gene, organism = "human", pvalueCutoff =0.05,qvalueCutoff = 0.05) ##小鼠mmu

write.table(kk,file="kegg.txt",sep="\t",quote=F,row.names = F)

go=read.table("kegg.txt",sep = "\t",header = T,check.names = F)

mytheme<- theme(axis.title = element_text(size = 13),

axis.text = element_text(size = 11),

plot.title = element_text(size = 14,

hjust= 0.5,

face= "bold"),

legend.title = element_text(size = 13),

legend.text = element_text(size = 11))

p2<- ggplot(data = go,

aes(x = Count,

y= Description))+

geom_point(aes(

color= -log10(qvalue)),size=6)+ # 气泡大小及颜色设置

theme_bw()+

scale_color_distiller(palette =1,direction = 1) +

labs(x = "Count",

y= "",

title= ""

) +xlim(5,13)+

mytheme

p2

###########Figure 5

#

library(forestplot)

rt=read.table("clipboard",header=T,sep="\t",row.names=1,check.names=F)

data=as.matrix(rt)

HR=data[,1:3]

hr=sprintf("%.3f",HR[,"HR"])

hrLow=sprintf("%.3f",HR[,"HR.95L"])

hrHigh=sprintf("%.3f",HR[,"HR.95H"])

pVal=data[,"pvalue"]

pVal=ifelse(pVal<0.001, "<0.001", sprintf("%.3f", pVal))

clrs=fpColors(box="red", line="darkblue", summary="royalblue")

tabletext <-

list(c(NA, rownames(HR)),

append("pvalue", pVal),

append("Hazard ratio",paste0(hr,"(",hrLow,"-",hrHigh,")")) )

pdf(file="forest.pdf", width=9, height=9, onefile=FALSE)

forestplot(tabletext,

rbind(rep(NA, 3), HR),

col=clrs,

graphwidth=unit(50, "mm"),

xlog=T,

lwd.ci=4,

boxsize=0.2,

title="Overall survival",

xlab="Hazard ratio",

txt_gp=fpTxtGp(ticks=gpar(cex=1.1), xlab=gpar(cex = 1.25))

)

dev.off()

#

library(limma)

rt=read.table("input.txt",sep="\t",header=T,check.names=F)

rt=as.matrix(rt)

rownames(rt)=rt[,1]

exp=rt[,2:ncol(rt)]

dimnames=list(rownames(exp),colnames(exp))

data=matrix(as.numeric(as.matrix(exp)),nrow=nrow(exp),dimnames=dimnames)

data=avereps(data)

library(ConsensusClusterPlus)

workDir=""

results = ConsensusClusterPlus(data,

maxK=9,

reps=100,

pItem=0.8,

pFeature=1,

title=workDir,

clusterAlg="km",

tmyPal = c("white","#A6CEE3"),

distance="euclidean",

seed=123456,

plot="png")

clusterNum=2

cluster=results[[clusterNum]][["consensusClass"]]

write.table(cluster,file="cluster.txt",sep="\t",quote=F,col.names=F)

#

library(limma)

library(ggplot2)

rt=read.table("input.txt",sep="\t",header=T,check.names=F)

rt=as.matrix(rt)

rownames(rt)=rt[,1]

exp=rt[,2:ncol(rt)]

dimnames=list(rownames(exp),colnames(exp))

data=matrix(as.numeric(as.matrix(exp)),nrow=nrow(exp),dimnames=dimnames)

data=avereps(data)

data=t(data)

cluster=read.table("cluster.txt",sep="\t",header=F)

cluster=as.vector(cluster[,2])

data.pca=prcomp(data)

pcaPredict=predict(data.pca)

PCA=data.frame(PC1=pcaPredict[,1], PC2=pcaPredict[,2],cluster=cluster)

PCA.mean=aggregate(PCA[,1:2], list(cluster=PCA$cluster), mean)

bioCol=c("#984EA3","#377EB8")

CluCol=bioCol[1:length(levels(factor(cluster)))]

veganCovEllipse<-function (cov, center = c(0, 0), scale = 1, npoints = 100) {

theta <- (0:npoints) * 2 * pi/npoints

Circle <- cbind(cos(theta), sin(theta))

t(center + scale * t(Circle %*% chol(cov)))

}

df_ell <- data.frame()

for(g in levels(factor(PCA$cluster))){

df_ell <- rbind(df_ell, cbind(as.data.frame(with(PCA[PCA$cluster==g,],

veganCovEllipse(cov.wt(cbind(PC1,PC2),

wt=rep(1/length(PC1),length(PC1)))$cov,

center=c(mean(PC1),mean(PC2))))), cluster=g))

}

pdf(file="PCA.pdf", height=5, width=6.5)

ggplot(data = PCA, aes(PC1, PC2)) + geom_point(aes(color = cluster)) +

scale_colour_manual(name="Cluster", values =CluCol)+

theme_bw()+

theme(plot.margin=unit(rep(1.5,4),'lines'))+

geom_path(data=df_ell, aes(x=PC1, y=PC2, colour=cluster), size=1, linetype=2)+

annotate("text",x=PCA.mean$PC1, y=PCA.mean$PC2, label=PCA.mean$cluster, cex=7)+

theme(panel.grid.major = element_blank(), panel.grid.minor = element_blank())

dev.off()

#

library(survival)

library(survminer)

rt=read.table("survival input.txt",header=T,sep="\t",check.names=F,row.names=1)

rt$futime=rt$futime/365

diff=survdiff(Surv(futime, fustat) ~Cluster,data = rt)

pValue=1-pchisq(diff$chisq,df=1)

fit=survfit(Surv(futime, fustat) ~ Cluster, data = rt)

if(pValue<0.001){

pValue="<0.001"

}else{

pValue=paste0("=",round(pValue,3))

}

surPlot=ggsurvplot(fit,

data=rt,pval = TRUE,

conf.int=TRUE,

pval.size=5,

risk.table=T,

legend.labs=c("Cluster 1","Cluster 2"),

legend.title=c("Cluster"),

xlab="Time(years)",

ylab="Overall survival",

break.time.by = 1,

risk.table.title="",

palette=c("#984EA3","#377EB8"),

risk.table.height=.25)

pdf(file=paste0("Cluster-os.pdf"), width = 7, height = 6.5,onefile = FALSE)

print(surPlot)

dev.off()

#

field="Cluster"

flag1="Cluster 1"

flag2="Cluster 2"

rt=read.table("clinical-sym.txt",sep="\t",header=T,check.names=F)

trainFlag=rt[rt[,field]==flag1,]

trainFlag=cbind(trainFlag,flag="Cluster 1")

testFlag=rt[rt[,field]==flag2,]

testFlag=cbind(testFlag,flag="Cluster 2")

newTable=rbind(trainFlag,testFlag)

newLabels=c("id")

for(i in 2:(ncol(rt)-1) ){

nameStat=colnames(newTable)[i]

tableStat=table(newTable[,c(nameStat,"flag")])

pStat=chisq.test(tableStat,correct = T)

pvalue=pStat$p.value

if(pvalue<0.001){

newLabels=c(newLabels,paste0(colnames(newTable)[i],"***"))

}else if(pvalue<0.01){

newLabels=c(newLabels,paste0(colnames(newTable)[i],"**"))

}else if(pvalue<0.05){

newLabels=c(newLabels,paste0(colnames(newTable)[i],"*"))

}else{

newLabels=c(newLabels,colnames(newTable)[i])

}

print(paste(colnames(newTable)[i],pvalue,sep=" "))

}

newLabels=c(newLabels,colnames(newTable)[ncol(rt)])

colnames(rt)=newLabels

write.table(rt,file="clusterCliGroup.Sig.txt",sep="\t",row.names=F,quote=F)

rt=read.table("input-clin.txt",sep="\t",header=T,row.names=1,check.names=F) #读取文件

outpdf="clin-heatmap.pdf"

library(pheatmap)

Type=read.table("clusterCliGroup.Sig.txt",sep="\t",header=T,row.names=1,check.names=F)

Type=Type[order(Type$Cluster),] #使clusterCliGroup.Sig按cluster列排序，cluster1在前cluster2在后，方面热图注释

rt=rt[,row.names(Type)] #使clusterCliExp.txt列名与clusterCliGroup.Sig行名，即两表格的样本排序一致

pdf(outpdf,height=5,width=15)

pheatmap(rt, annotation=Type, #"#A6CEE3"浅蓝 "#1F78B4"宝石蓝 "#B2DF8A"浅绿 "#33A02C"绿 "#FB9A99"浅玫红 "#E31A1C"大红 "#FDBF6F"渚黄 "#FF7F00"橙 "#CAB2D6"浅紫 "#6A3D9A"大紫 "#FFFF99"深米黄 "#B15928"

annotation_colors =list(Gender=c("Female"="#66C2A5","Male"="#FC8D62"),Mstage=c("M0"="#8DA0CB","M1"="#E78AC3","MX"="#A6D854"),Nstage=c("N0"="#FFD92F","N1"="#E5C494","N2"="#B3B3B3","N3/NX"="#8DD3C7"),Tstage=c("T1"="#FFFFB3","T2"="#BEBADA","T3"="#FB8072","T4"="#80B1D3"),Histologic_grade=c("G1"="#FDB462","G2"="#B3DE69","G3"="#FCCDE5","GX"="#D9D9D9"),Pathologic_stage=c("Stage I"="#BC80BD","Stage II"="#CCEBC5","Stage III"="#FFED6F","Stage IV"="#A6CEE3"),Cluster=c("Cluster 1"="#984EA3","Cluster 2"="#377EB8"),New_tumor_events=c("YES"="#33A02C","NO"="#FB9A99")),

color = colorRampPalette(c("#984EA3", "white", "#377EB8"))(50),

cluster_cols =F,

fontsize=8,

fontsize_row=8,

scale="row",

show_colnames=F,

fontsize_col=3)

dev.off()

############Figure 6

#

library(limma)

library(pheatmap)

riskFile="cluster.txt"

immFile="TIMER2.0.txt"

risk=read.table(riskFile, header=T, sep="\t", check.names=F, row.names=1)

immune=read.table(immFile, header=T, sep="\t", check.names=F, row.names=1)

immune=as.matrix(immune)

immune=avereps(immune)

sameSample=intersect(row.names(risk), row.names(immune))

#risk=risk[sameSample, c("risk", "riskScore")]

immune=immune[rownames(risk),]

data=cbind(risk, immune)

outTab=data.frame()

sigCell=c("Cluster")

for(i in colnames(data)[2:ncol(data)]){

if(sd(data[,i])<0.001){next}

wilcoxTest=t.test(data[,i] ~ data[,"Cluster"])

pvalue=wilcoxTest$p.value

if(wilcoxTest$p.value<0.05){

outTab=rbind(outTab,cbind(immune=i, pvalue))

sigCell=c(sigCell, i)

}

}

write.table(file="immuneCor.txt", outTab, sep="\t", quote=F, row.names=F)

data=data[,sigCell]

data=data[order(data[,"Cluster"]),]

annCol=as.data.frame(data[,1])

colnames(annCol)=c("Cluster")

rownames(annCol)=rownames(data)

annCol[,"Cluster"]=factor(annCol[,"Cluster"], unique(annCol[,"Cluster"]))

data=t(data[,(2:ncol(data))])

annRow=sapply(strsplit(rownames(data),"_"), '[', 2)

annRow=as.data.frame(annRow)

row.names(annRow)=row.names(data)

colnames(annRow)=c("Methods")

annRow[,"Methods"]=factor(annRow[,"Methods"], unique(annRow[,"Methods"]))

gapCol=as.vector(cumsum(table(annCol[,"Cluster"])))

gapRow=as.vector(cumsum(table(annRow[,"Methods"])))

Cluster=c("#984EA3", "#377EB8")

names(Cluster)=c("Cluster 1", "Cluster 2")

ann_colors=list(Cluster=Cluster)

pdf("immHeatmap.pdf", width=7, height=6)

pheatmap(data,

annotation_col =annCol,

annotation_row=annRow,

annotation_colors = ann_colors,

color = colorRampPalette(c(rep("#984EA3",5), "white", rep("#377EB8",5)))(100),

cluster_cols =F,

cluster_rows =F,

gaps_row=gapRow,

gaps_col=gapCol,

scale="row",

show_colnames=F,

show_rownames=T,

fontsize=6,

fontsize_row=5,

fontsize_col=6)

dev.off()

#

library(tidyverse)

library(ggpubr)

Type=read.table("cluster.txt",sep="\t",check.names=F,row.names=1,header=T)

rt=read.table("ICI.txt",sep="\t",check.names=F,row.names=1,header=T)

Type=Type[colnames(rt),]

rt=t(rt)

data=data.frame()

for(i in colnames(rt)){

data=rbind(data,cbind(expression=(rt[,i]),gene=i,Cluster=as.vector(Type)))

}

write.table(data,file="data.txt",sep="\t",quote=F)

df=read.table("data.txt",sep = "\t",header = T,row.names = 1,check.names = F)

ggplot(df,aes(x = gene, y = expression,fill = Cluster)) +

geom_boxplot(width = .5,show.legend = F,

position = position_dodge(0.9),

color = 'grey20',alpha = 0.5,

outlier.color = 'grey50') +

geom_violin(position = position_dodge(0.9),alpha = 0.5,

width = 2.5,trim = T,

color = NA) +

theme_bw() +

theme(axis.text.x = element_text(angle = 45,hjust = 1,color = 'black'),

legend.position = 'top') +xlab("")+ylab("Expression")+

scale_fill_manual(values = c('Cluster 1'='#FB8072','Cluster 2'='#A6CEE3'),

name = '') +

stat_compare_means(aes(group=Cluster),

symnum.args=list(cutpoints = c(0, 0.001, 0.01, 0.05, 1),

symbols = c("***", "**", "*", "")),label = "p.signif",

label.y = 12,size = 4.5)

#########Figure 7

#

conNum=84

treatNum=99

library(limma)

rt=read.table("sampleExp.txt",sep="\t",header=T,check.names=F)

rt=as.matrix(rt)

rownames(rt)=rt[,1]

exp=rt[,2:ncol(rt)]

dimnames=list(rownames(exp),colnames(exp))

rt=matrix(as.numeric(as.matrix(exp)),nrow=nrow(exp),dimnames=dimnames)

rt=avereps(rt)

rt=rt[rowMeans(rt)>0,]

rt=normalizeBetweenArrays(as.matrix(rt))

modType=c(rep("low",conNum),rep("high",treatNum))

design <- model.matrix(~0+factor(modType))

colnames(design) <- c("high","low")

fit <- lmFit(rt,design)

cont.matrix<-makeContrasts(high-low,levels=design)

fit2 <- contrasts.fit(fit, cont.matrix)

fit2 <- eBayes(fit2)

allDiff=topTable(fit2,adjust='fdr',number=200000)

write.table(allDiff,file="diff-All.txt",sep="\t",quote=F)

#

library(org.Hs.eg.db)

library(clusterProfiler)

library(pathview)

library(enrichplot)

library(dplyr)

data <- read.table("diff-All.txt",header=TRUE,check.names = F,sep = "\t")

gene <- data$SYMBOL

gene=bitr(gene,fromType="SYMBOL",toType="ENTREZID",OrgDb="org.Hs.eg.db")

gene <- dplyr::distinct(gene,SYMBOL,.keep_all=TRUE)

data_all <- data %>%

inner_join(gene,by="SYMBOL")

data_all_sort <- data_all %>%

arrange(desc(logFC))

geneList = data_all_sort$logFC

names(geneList) <- data_all_sort$ENTREZID

kegg_gmt <- read.gmt("h.all.v2022.1.Hs.entrez.gmt")

gsea <- GSEA(geneList,

TERM2GENE = kegg_gmt) #GSEA分析

write.table(gsea,"GSEA-HALLMARK.txt",sep="\t",row.names=F,quote=F)

pdf(file="GSEA-HALLMARK_MYC_TARGETS_V2.pdf",width=10,height=9)

gseaplot2(gsea,

"HALLMARK_MYC_TARGETS_V2",

pvalue_table = F,

subplots = 1:3,

base_size=21)

dev.off()

#

inputFile="tpm-T-surlog.txt"

gmtFile="c2.cp.kegg.v2022.1.Hs.symbols.gmt"

library(GSVA)

library(limma)

library(GSEABase)

rt=read.table(inputFile,sep="\t",header=T,check.names=F)

rt=as.matrix(rt)

rownames(rt)=rt[,1]

exp=rt[,2:ncol(rt)]

dimnames=list(rownames(exp),colnames(exp))

mat=matrix(as.numeric(as.matrix(exp)),nrow=nrow(exp),dimnames=dimnames)

mat=avereps(mat)

mat=normalizeBetweenArrays(mat)

c3gsc2=getGmt( gmtFile,

collectionType=BroadCollection(category="c3"),

geneIdType=SymbolIdentifier())

gsvaOut=gsva(mat,

c3gsc2,

min.sz=10,

max.sz=500,

verbose=TRUE,

parallel.sz=1)

gsvaOut=rbind(id=colnames(gsvaOut),gsvaOut)

write.table(gsvaOut,file="gsvaOut.txt",sep="\t",quote=F,col.names=F)

library(pheatmap)

conNum=84

treatNum=99

rt=read.table("gsvaOut-sig.txt",sep = "\t",row.names = 1,check.names = F,header = T)

Cluster=c(rep("Cluster 1",conNum),rep("Cluster 2",treatNum))

names(Cluster)=colnames(rt)

Cluster=as.data.frame(Cluster)

pdf(file="Heatmap-kegg.pdf",height=10,width=9)

pheatmap(rt,

annotation=Cluster,

annotation_colors = list(Cluster=c("Cluster 1"="#984EA3","Cluster 2"="#377EB8")),

color = colorRampPalette(c("#984EA3", "white", "#377EB8"))(100), ####"blue3", "white", "yellow2"

cluster_cols =F,

border=F,

show_colnames = F,

show_rownames = T,

scale="row",

fontsize = 10,

fontsize_row=10,

fontsize_col=8)

dev.off()

#############Figure 8

library(limma)

library(ggpubr)

library(pRRophetic)

library(ggplot2)

set.seed(12345)

expFile="tpm-T-surlog.txt"

riskFile="cluster.txt"

allDrugs=c("AICAR", "AKT.inhibitor.VIII", "ATRA","Axitinib", "AZD.0530", "AZD.2281", "AZD6244", "AZD6482", "AZD7762", "AZD8055", "Bexarotene","Bicalutamide", "Bleomycin", "Bortezomib", "Bosutinib", "Camptothecin", "Cisplatin", "CMK", "Cyclopamine", "Cytarabine", "Dasatinib", "Docetaxel", "Doxorubicin", "Elesclomol", "Embelin", "Epothilone.B", "Erlotinib", "Etoposide", "Gefitinib", "Gemcitabine", "Imatinib", "JNK.Inhibitor.VIII","Lapatinib", "Lenalidomide", "Metformin", "Methotrexate", "Midostaurin", "Nilotinib", "Paclitaxel", "Parthenolide", "Pazopanib", "Pyrimethamine", "Rapamycin","Roscovitine", "Salubrinal", "Sorafenib", "S.Trityl.L.cysteine", "Sunitinib", "Temsirolimus", "Thapsigargin", "Tipifarnib", "Vinblastine", "Vinorelbine", "Vorinostat")

rt = read.table(expFile, header=T, sep="\t", check.names=F)

rt=as.matrix(rt)

rownames(rt)=rt[,1]

exp=rt[,2:ncol(rt)]

dimnames=list(rownames(exp),colnames(exp))

data=matrix(as.numeric(as.matrix(exp)),nrow=nrow(exp),dimnames=dimnames)

data=avereps(data)

data=data[rowMeans(data)>0.5,]

riskRT=read.table(riskFile, header=T, sep="\t", check.names=F, row.names=1)

for(drug in allDrugs){

senstivity=pRRopheticPredict(data, drug, selection=1)

senstivity=senstivity[senstivity!="NaN"]

sameSample=intersect(row.names(riskRT), names(senstivity))

Cluster=riskRT[sameSample, "Cluster",drop=F]

senstivity=senstivity[sameSample]

rt=cbind(Cluster, senstivity)

rt$Cluster=factor(rt$Cluster, levels=c("Cluster 1", "Cluster 2"))

type=levels(factor(rt[,"Cluster"]))

comp=combn(type, 2)

my_comparisons=list()

for(i in 1:ncol(comp)){my_comparisons[[i]]<-comp[,i]}

test=wilcox.test(senstivity~Cluster, data=rt)

if(test$p.value<0.05){

boxplot=ggboxplot(rt, x="Cluster", y="senstivity", fill="Cluster",

xlab="Cluster",

ylab=paste0(drug, " senstivity (IC50)"),

legend.title="Cluster",

palette=c("#984EA3","#377EB8")

)+

stat_compare_means(comparisons=my_comparisons, method="t.test",symnum.args=list(cutpoints = c(0, 0.001, 0.01, 0.05, 1), symbols = c("***", "**", "*", "ns")), label = "p.signif")

pdf(file=paste0("durgSenstivity.", drug, ".pdf"), width=5, height=4.5)

print(boxplot)

dev.off()

}

}

###########Figure 9

#

library(corrplot) #引用包

rt=read.table("input.txt",sep="\t",header=T,row.names=1,check.names=F) #读取输入文件

pdf("corrplot.pdf",height=7,width=7) #保存图片的文件名称

par(oma=c(0.5,1,1,1.2))

M=cor(t(rt))

corrplot(M, order = "AOE", type = "upper", tl.pos = "lt")

corrplot(M, add = TRUE, type = "lower", method = "number", order = "AOE",

col = "black", diag = FALSE, tl.pos = "n", cl.pos = "n")

dev.off()

#

inputFile="cnvMatrix.txt" #输入文件

rt=read.table(inputFile, header=T, sep="\t", check.names=F, row.names=1) #读取输入文件

GAIN=rowSums(rt> 0) #拷贝数增加的样品数目

LOSS=rowSums(rt< 0) #拷贝数缺失的样品数目

GAIN=GAIN/ncol(rt)*100 #拷贝数增加的百分率

LOSS=LOSS/ncol(rt)*100 #拷贝数缺失的百分率

data=cbind(GAIN, LOSS)

data=data[order(data[,"GAIN"],decreasing = T),]

#绘制图形

data.max = apply(data, 1, max)

pdf(file="CNVfreq.pdf", width=7, height=5)

cex=1.3

par(cex.lab=cex, cex.axis=cex, font.axis=2, las=1, xpd=T)

bar=barplot(data.max, col="grey80", border=NA,

xlab="", ylab="CNV.frequency(%)", space=1.5,

xaxt="n", ylim=c(0,1.2*max(data.max)))

points(bar,data[,"GAIN"], pch=20, col="#E41A1C", cex=3)

points(bar,data[,"LOSS"], pch=20, col="#377EB8", cex=3)

legend("top", legend=c('GAIN','LOSS'), col=c("#E41A1C","#377EB8"), pch=20, bty="n", cex=2, ncol=2)

par(srt=45)

text(bar, par('usr')[3]-0.2, rownames(data), adj=1)

dev.off()

#

library("RCircos") #引用包

#初始化圈图

cytoBandIdeogram=read.table("refer.txt", header=T, sep="\t")

chr.exclude <- NULL

cyto.info <- cytoBandIdeogram

tracks.inside <- 5

tracks.outside <- 0

RCircos.Set.Core.Components(cyto.info, chr.exclude, tracks.inside, tracks.outside)

#设置圈图参数

rcircos.params <- RCircos.Get.Plot.Parameters()

rcircos.params$text.size=1

rcircos.params$point.size=5

RCircos.Reset.Plot.Parameters(rcircos.params)

#输出文件

pdf(file="RCircos.pdf", width=8, height=8)

RCircos.Set.Plot.Area()

RCircos.Chromosome.Ideogram.Plot()

#散点图

RCircos.Scatter.Data=read.table("Rcircos.scatter.txt", header=T, sep="\t", check.names=F)

data.col <- 4

track.num <- 1

side <- "in"

RCircos.Scatter.Plot(RCircos.Scatter.Data, data.col, track.num, side, by.fold=0.1)

#加上基因名称

RCircos.Gene.Label.Data=read.table("Rcircos.geneLabel.txt", header=T, sep="\t", check.names=F)

name.col <- 4

side <- "in"

track.num <- 2

RCircos.Gene.Connector.Plot(RCircos.Gene.Label.Data, track.num, side)

track.num <- 3

RCircos.Gene.Name.Plot(RCircos.Gene.Label.Data, name.col, track.num, side)

dev.off()

#
